# Mechanochemical Synthesis of a Sodium Anion Complex [Na^+^(2,2,2-cryptand)Na^–^] and Studies of Its Reactivity: Two-Electron and One-Electron Reductions

**DOI:** 10.1021/acs.inorgchem.4c02914

**Published:** 2024-07-29

**Authors:** Nathan Davison, Jack M. Hemingway, Corinne Wills, Tomislav Stolar, Paul G. Waddell, Casey M. Dixon, Luke Barron, James A. Dawson, Erli Lu

**Affiliations:** † School of Chemistry, University of Birmingham, Edgbaston, Birmingham B15 2TT, U.K.; ‡ Chemistry, School of Natural and Environmental Sciences, Newcastle University, Newcastle upon Tyne NE1 7RU, U.K.; § Federal Institute for Materials Research and Testing (BAM), 12489 Berlin, Germany

## Abstract

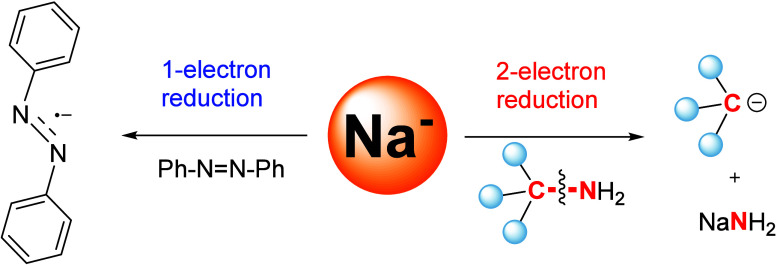

Group 1 metal molecular chemistry is dominated by a +1 oxidation state, while a 0 oxidation state is widespread in the metals. A more exotic, yet still available, oxidation state of group 1 metal is −1, i.e., alkalide. Reported as early as the 1970s, the alkalides appear in every modern inorganic chemistry textbook as an iconic chemical curiosity, yet their reactivity remains unexplored. This is due to their synthetic hurdles. In this work, we report the first facile synthesis of the archetypical alkalide complex, [Na^+^(2,2,2-cryptand)Na^–^], which allows us to unveil a versatile reactivity profile of this once exotic species.

## Abstract 2

Group 1 metal anions, i.e., alkalides, have been textbook iconic chemical curiosities since their landmark, yet technically demanding, synthesis reported first in the 1970s. Despite being known for decades, the reactivity of alkalides has been barely studied due to their poor accessibility. Herein, we report the first facile synthesis of the archetypical alkalide, a sodium sodide [Na^+^(2,2,2-cryptand)Na^−^] (**1**), enabled by mechanochemical ball milling. Studies of the reactivity of **1** unveil a versatile profile, including one-electron and two-electron reductions.

## Introduction

**Figure 1:**
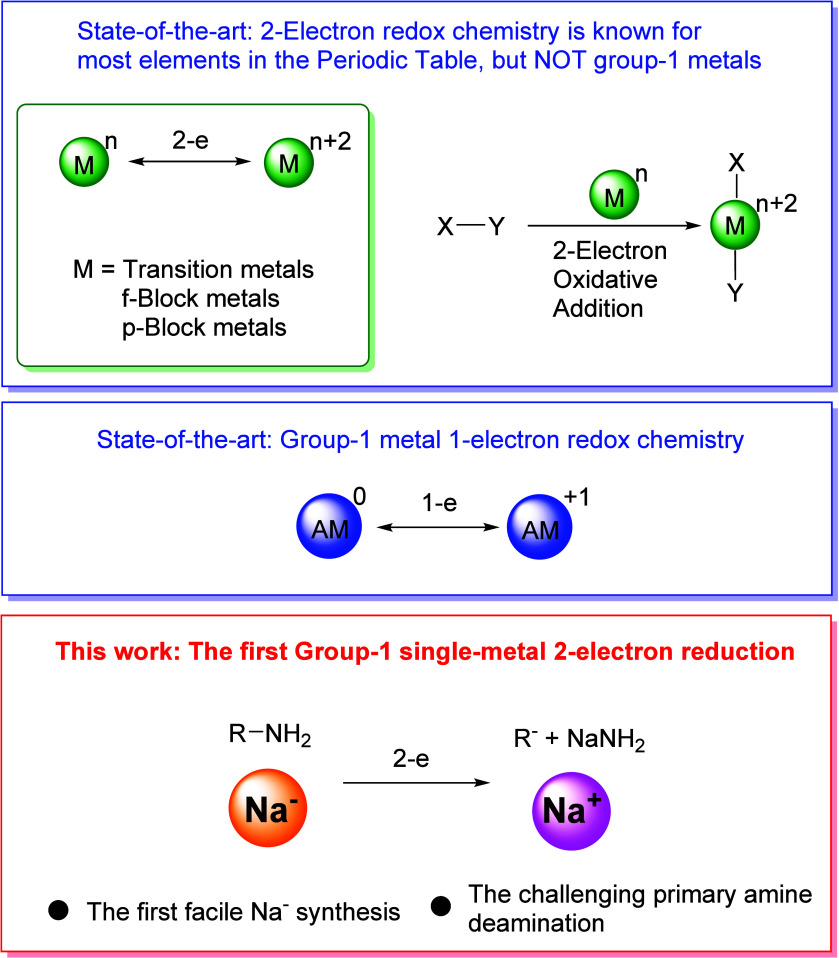
State of the art and this work. Two-electron redox chemistry is known for d-, p-, and f-block metals, but not for group 1 alkali metals (top). State-of-the-art group 1 metal redox chemistry (middle) and this work (bottom).

Reduction and oxidation (redox) reactions involve interconversions between an element’s available oxidation states and are the cornerstones of organometallic catalysis, such as oxidative addition and reductive elimination. An element’s redox chemistry is underpinned by its available oxidation states. For example, the Pd(0) ↔ Pd(+2) interconversion is the foundation of the classic Suzuki–Miyaura coupling.^1^ Such single-metal two-electron redox processes are traditionally limited to transition metals, while recent efforts have expanded the scope to lanthanide,^2^ actinide,^3,4^ and p-block (groups 13–15^5^) (Figure 1) metals, as well as non-metal centers such as phosphorus.^6^ From a sustainability perspective, developing main-group metal-mediated two-electron redox processes is crucially important for developing future sustainable catalysts and has hence attracted significant and fast-growing interest across the inorganic, organic, and catalysis communities.^5^

Group 1 alkali metals (AMs) are among the most abundant elements in the Earth’s crust (e.g., Na has an abundance of 2.6%). There are three available oxidation states for the group 1 metals: +1, 0, and −1. While their molecular chemistry is exclusively in the +1 oxidation state, the one-electron AM(0) ↔ AM(+1) process plays essential roles in chemical synthesis as widely used reductants (e.g., potassium mirror and potassium graphite) as well as in batteries. Hitherto, single-metal two-electron processes, i.e., AM(−1) ↔ AM(+1), are unknown for the alkali metals, which is due to poor accessibility to an iconic chemical curiosity, the long-known yet surprisingly underdeveloped AM(−1) anions, i.e., the alkalides.

Since the first reports in the 1970s by the group of the late James Dye,^7−9^ alkalides appear in every inorganic chemistry textbook.^10^ Discussions about the nature of the exotic −1 oxidation-state alkali metal anions have continued until today,^11,12^ along with several *in silico* theoretical studies from the physics and physical chemistry communities.^13−16^ Nonetheless, from a synthetic chemistry perspective, the reactivity of alkalides, including their potential in two-electron redox chemistry, has never been properly studied apart from a few decomposition studies.^17,18^ This *status quo* is due to their formidable synthetic hurdles.

The classical alkalide synthetic methods require technically demanding conditions, such as extremely volatile solvents (e.g., methylamine), specially designed glassware, and unconventional synthetic protocols (e.g., metal vapor deposition under 10^–5^ Torr high vacuum), and suffer from poor scalability and low yields.^19−23^

Herein, we overcome the alkalide synthetic hurdles by introducing a mechanochemical ball milling method.^24,25^ The new protocol allows us to synthesize the archetypical alkalide, a sodide [Na^+^(2,2,2-cryptand)Na^–^] (**1**),^8^ from readily available starting materials (sodium metal and 2,2,2-cryptand) at a millimole scale in reasonable and reproducible yield (≤48%). We characterize **1** as the modern standard and clarify several historical ambiguities and/or inaccuracies, especially with regard to ^23^Na nuclear magnetic resonance (NMR) and single-crystal X-ray diffraction (SCXRD) studies, as well as its electronic structure via quantum chemical density functional theory (DFT) calculations. More importantly, we find that **1** exhibits a versatile reactivity profile, including the first alkali metal-mediated single-metal two-electron process (C–N cleavage deamination in primary amines), and also a one-electron reduction toward azobenzene. The findings are elaborated in the following sections.

## Results and Discussion

**Figure 2:**
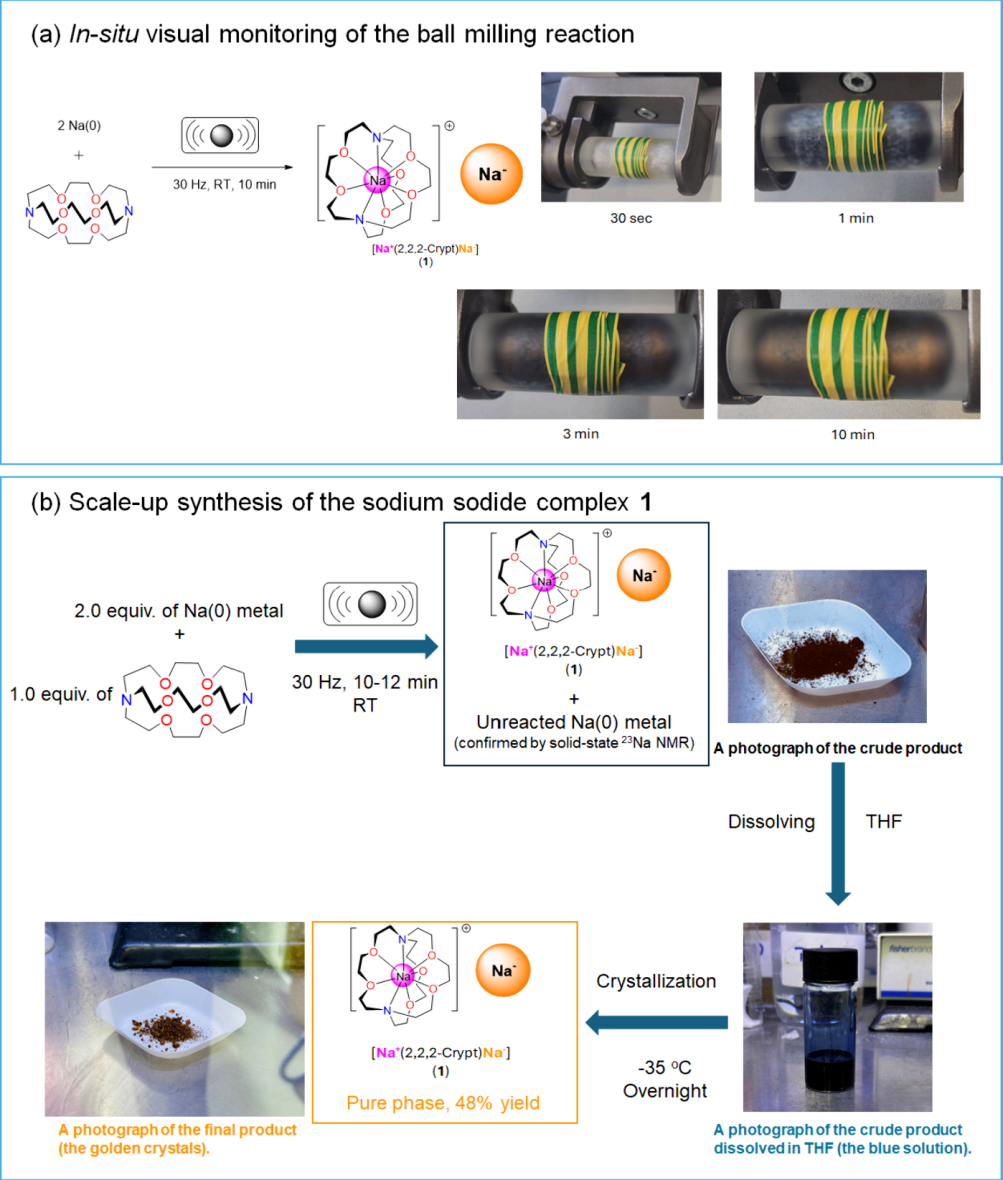
Mechanochemical synthesis of **1**. (a) *In situ* visual monitoring of the ball milling reaction between Na metal and 2,2,2-cryptand in a transparent PMMA jar. (b) Synthesis and crystallization of sodide complex **1**.

Inspired by recent success in the use of mechanochemical ball milling in s-block chemistry,^24,26−28^ we tested a reaction between 2 equiv of sodium metal and 1 equiv of 2,2,2-cryptand. The ball milling reaction was first monitored in a 14 mL transparent poly(methyl methacrylate) (PMMA) jar under argon, at room temperature, at a frequency of 30 Hz. The starting materials were initially converted into a deep blue mixture within 1 min of milling and then into a golden substance within 10 min (Figure 2a). The fleet intermediate deep blue color indicates an interesting possibility: an electride intermediate was formed at the initial stage of milling, i.e., Na(0) + 2,2,2-cryptand → [Na^+^(2,2,2-cryptand)e^–^], which subsequently reduces Na(0) to Na^–^, i.e., [Na^+^(2,2,2-cryptand)e^–^] + Na(0) → [Na^+^(2,2,2-cryptand)Na^–^].

**Figure 3:**
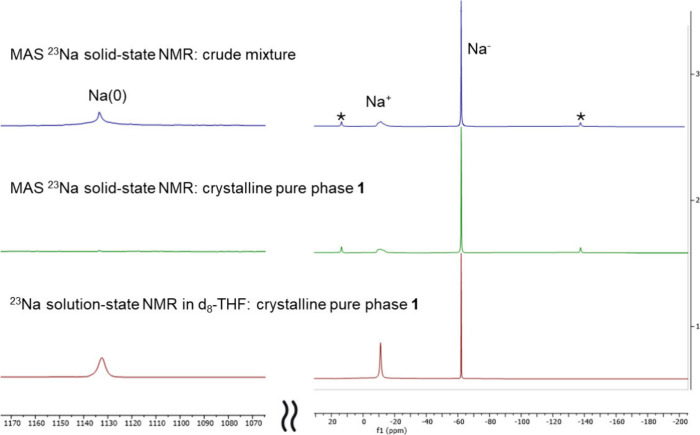
Solid-state and solution ^23^Na NMR studies of **1** as the crude product and pure phase. Magic angle spinning (MAS) solid-state ^23^Na NMR spectra of the crude mixture (top) and the pure phase of **1** (middle) and solution-state ^23^Na NMR spectrum of the pure phase of **1** in *d*_8_-THF (bottom). All of the NMR spectra were collected at 298 K. *MAS spinning sidebands.

On the basis of visual observation, the preparative scale reaction condition was set as 30 Hz for 10 min at room temperature. A black-brown solid crude product was produced (Figure 2b), which was proven to be a mixture of target sodide **1** and unreacted Na(0) metal by a magic angle spinning (MAS) solid-state ^23^Na NMR spectrum (Figure 3 top). The two signals at −11 and −62 ppm represent Na^+^ and Na^–^ of **1**, respectively. The chemical shifts of these two signals match Dye’s reports in three solvents [tetrahydrofuran (THF), ethylamine, and methylamine]^29^ and those from a single-crystal ^23^Na NMR study, as well.^30,31^ The Na^–^ signal of **1** in Figure 3 also matches a recent report of a potassium sodide complex in solution by Barrett et al. (^23^Na NMR chemical shifts −61 to −63 ppm),^32^ despite crystalline material not being obtained therein. The Dye group and others conducted ^23^Na NMR studies of **1** and other related sodides^33−35^ and reported a sodium metal Na(0) contamination at 1120 ppm.^22c^ The chemical shift matches the results of recent theoretical^36^ and experimental^37^^23^Na NMR studies on the zerovalent metallic Na(0) species. Hence, we expanded our scanning range of the MAS ^23^Na NMR for the crude product and indeed located a signal at 1134 ppm (Figure 3, top), which can be attributed to unreacted Na(0) metal, matching that which Gray and co-workers reported in their MAS ^23^Na NMR studies in the Na-ion battery context.^36^ Hence, we can conclude that the crude product from the ball milling is a mixture of **1**, unreacted Na(0) metal, and potentially unreacted 2,2,2-cryptand.

Pure **1** was obtained as golden/bronze crystals from a THF solution of the crude mixture after it had been kept at −35 °C overnight, and its SCXRD structure was again investigated. The crystals were found to be similar in appearance to those described by Dye et al. in their 1974 original report,^8^ but our crystallization procedure is much simpler (THF left to stand in a −35 °C freezer vs slow evaporation and multiple washings with ethylamine at a cryogenic temperature). The bulk golden crystals were found to be thin flakes, as previously described in ref (9), and could be mounted on the goniometer of an X-ray diffractometer with little difficulty.

Whereas the structure of **1** in Dye’s original report in 1974 (Cambridge Structural Database deposition reference CRYPNA10)^9^ was determined with space group *R*3_2_, we posit that the space group is more likely to be *I*2. The structure determined from the data reported here does solve in *R*3_2_ but with much higher refinement metrics (e.g., *R*_1_ = 6.80%, and *wR*_2_ = 21.39%). Determining the structure in monoclinic space group *I*2 provides much better agreement with the data. Full crystallography details can be found in Table S1 (our new solution) and Table S2 (the previous solution), while the refined molecular structure of **1** can be found in Figure S22. The sodide anion, Na2, is found isolated from the rest of the structure. The Na^–^–Na^+^ distances from a sodide anion to its neighboring Na^+^ cations are ∼7.0 Å. The anion–anion distances range from ∼8.7 to ∼10.9 Å, indicating that there are no anion–anion inter-alkalide interactions, which have been observed in some other sodides.^22c^

Once isolated, the golden crystals of **1** can be handled at room temperature for at least 12 h, allowing further characterization. The MAS ^23^Na solid-state NMR study of the golden crystals confirmed that it is a pure phase of **1** (Figure 3, middle). While the −11 and −62 ppm signals persist, the 1134 ppm signal disappeared, indicating that the unreacted Na(0) metal was successfully removed. The crystalline yield of **1** was 48% at a 2 mmol scale (see Experimental Section at the end of the paper).

**Figure 4:**
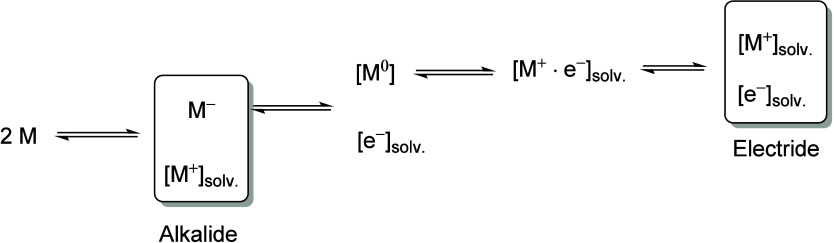
Equilibria between the alkalide and electride phases.

**1** is insoluble in hexane and immediately reacts with benzene at room temperature, where the characteristic golden color disappears and an off-white suspension with intractable ^1^H/^23^Na NMR spectra is formed. Dissolving **1**’s crystals in *d*_8_-THF resulted in a deep blue solution, which gave a surprising ^23^Na NMR spectrum. The 1134 ppm signal of Na(0) reappears (Figure 3, bottom). The resurgence of the Na(0) signal in the THF solution may appear to be confusing, but it has been hypothesized for a long time that an alkalide can undergo a series of equilibria (Figure 4);^33e,33f^ this hypothesis was reported even before the discovery of the first crystalline alkalide **1**.^38^ This equilibrium hypothesis was also supported by modern thermodynamic cycle calculations.^39^ We hypothesize that such an equilibrium in the THF solution of **1** leads to the observed Na(0) signal.

Once isolated as the golden crystals, **1** is stable at room temperature under argon for at least 12 h, allowing the following reactivity studies (*vide infra*). Storage at room temperature for a few days will eventually lead to decolorization of the golden crystals, which is a sign of decomposition. With regard to **1**’s thermal stability in a THF solution, we found that the rate of decolorization (decomposition) of the blue THF solution depends on the concentration. The more dilute the solution, the faster the decomposition, indicating the major decomposition pathway is reaction with the solvent THF, which corresponds well with the previous alkalide decomposition studies.^17,18^ Whereas at a concentration of 0.25 mmol/L, the solution decolorized almost immediately (which prevented us from obtaining an ultraviolet–visible absorption spectrum), a NMR concentration (∼0.1 mol/L in *d*_8_-THF) solution was found to be stable at room temperature for ∼2 h before eventually fading into a colorless solution (^1^H NMR, only 2,2,2-cryptand; ^23^Na NMR, only Na^+^ species). We also conducted variable-temperature ^23^Na NMR studies of **1** in *d*_8_-THF in the temperature range of 243–313 K (Figures S7 and S8) and monitored the decomposition of **1** in *d*_8_-THF (concentration of 80.5 mmol/L) at 313 K (Figures S9 and S10). The latter indicates that the Na(0) and Na^–^ signals disappear simultaneously upon decomposition, forming a Na^+^ species with a broad ^23^Na signal.

We conducted DFT calculations of **1**, which provide unprecedented insights into the electronic structure, especially the charge distribution and frontier molecular orbitals. A geometry optimization calculation well reproduced the SCXRD structure (see Section 2 of the Supporting Information for more detailed computational methods). On the basis of the optimized structure, we conducted natural bonding orbital (NBO) charge analysis, which is the most effective diagnostic parameter for distinguishing between a Na^+^ and a Na^–^. The NBO charge on Na^–^ was calculated as −0.880, in sharp contrast with the value of +0.823 on the Na^+^. The NBO charges unquestionably confirm the sodide nature of **1**. We also calculated the isotropic NMR shielding tensors (in parts per million) of Na^+^ and Na^–^, which are 569.8745 and 629.8618 ppm, respectively. The relationship between the NMR shielding tensor (**σ**) and the observed chemical shift (δ) is defined by Facelli^40^ in the equation δ = *a*σ_iso_ – **σ**, where *a* is the unit matrix and σ_iso_ is the isotropic value or trace of the shielding tensor of a certain element’s (here ^23^Na) standard NMR reference. The difference between the calculated NMR shielding tensor of the Na^+^ and Na^–^ centers in **1** agrees well with the experimentally observed chemical shift difference, as well as with the literature precedents.^31^

**Figure 5:**
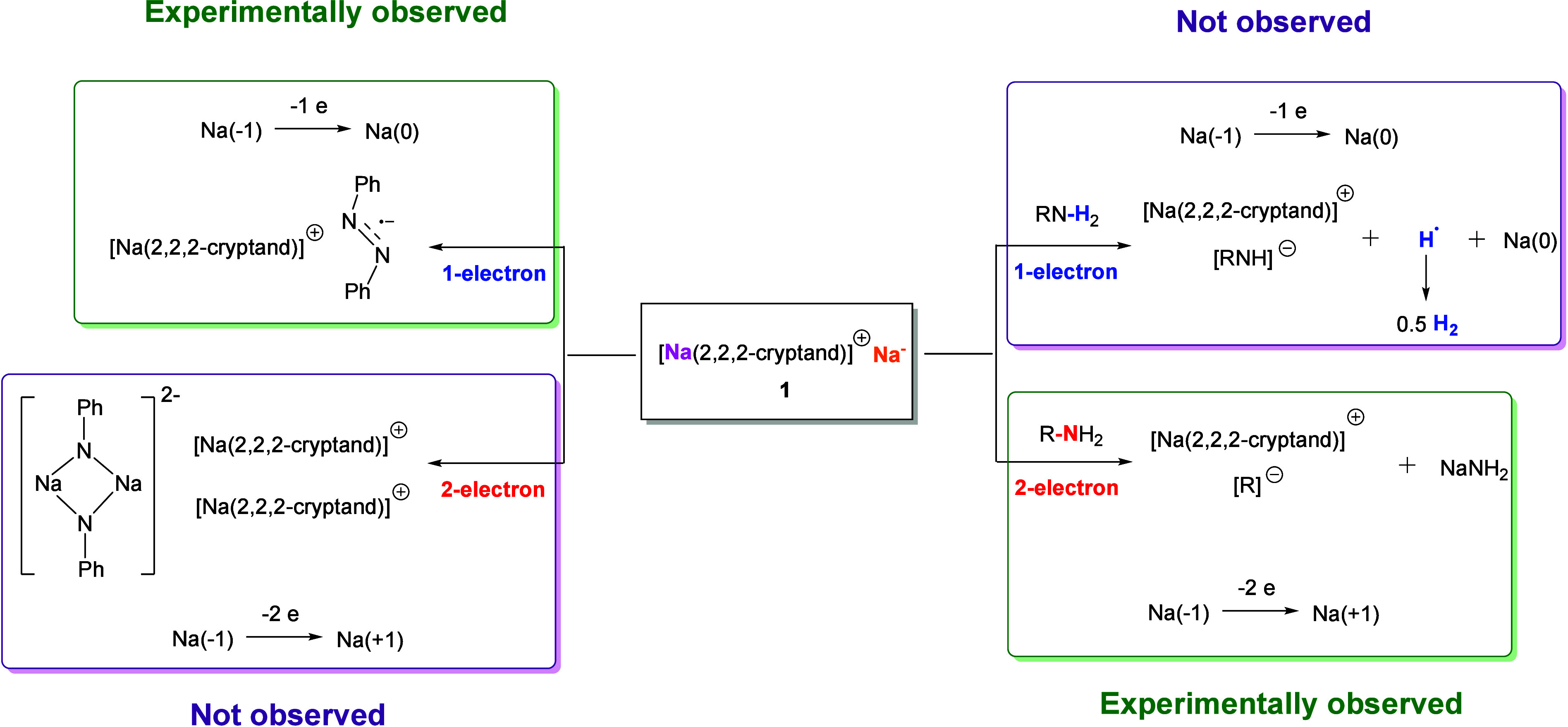
Possible one-electron and two-electron redox reaction pathways of **1**. Reactions between **1** and primary amines (right) or azobenzene (left) could take one-electron or two-electron pathways, but only one pathway is experimentally observed for each: the two-electron pathway for primary amines and the one-electron pathway for azobenzene.

The improved access to **1** enabled by the ball milling method unlocks the gateway to test its reactivity. In principle, the Na^–^ center in **1** can conduct both one-electron [Na(−1) → Na(0)] and two-electron [Na(−1) → Na(+1)] reductions. We chose two classes of substrates to assess the one-electron versus two-electron reactivity: primary amines and azobenzene (Figure 5). Primary amines have two reactive sites: the N–H bond and the C–N bond. Among the two sites, the Brønsted acidic N–H bond is reactive, labile to Brønsted acid–base deprotonation and one-electron reductive deprotonation. The latter was very recently reported by the Harder group in a case of electride-mediated one-electron reductive deprotonation.^41^ Along with the one-electron N–H reductive deprotonation, two-electron N–H oxidative addition in primary amines was reported to be mediated by uranium^4^ and phosphorus^6a^ centers. In sharp contrast to the reactive N–H bond, the C–N bond in primary amines is much more inert and difficult to cleave. The poor electrophilicity of the -NH_2_ group renders the C–NH_2_ bond a challenging target for transition metal-catalyzed oxidative addition^42^ and reductive deamination,^43^ both of which are two-electron processes. As a footnote to the challenge, the organic synthesis community currently must convert the -NH_2_ into a better leaving group to conduct deamination, such as via isodiazene intermediates in a recent example,^44^ or using a strong Lewis acid catalyst such as B(C_6_F_5_)_3_ coupled with silanes as the reductant.^45^ With regard to azobenzene, it is a classic substrate for both one-electron and two-electron reductions. Noticeably, a bimetallic two-electron oxidative addition of azobenzene was recently reported to be facilitated by a U(+3) ↔ U(+5) interconversion, which demonstrated the presence of a two-electron process in uranium chemistry.^46^

**Figure 6:**
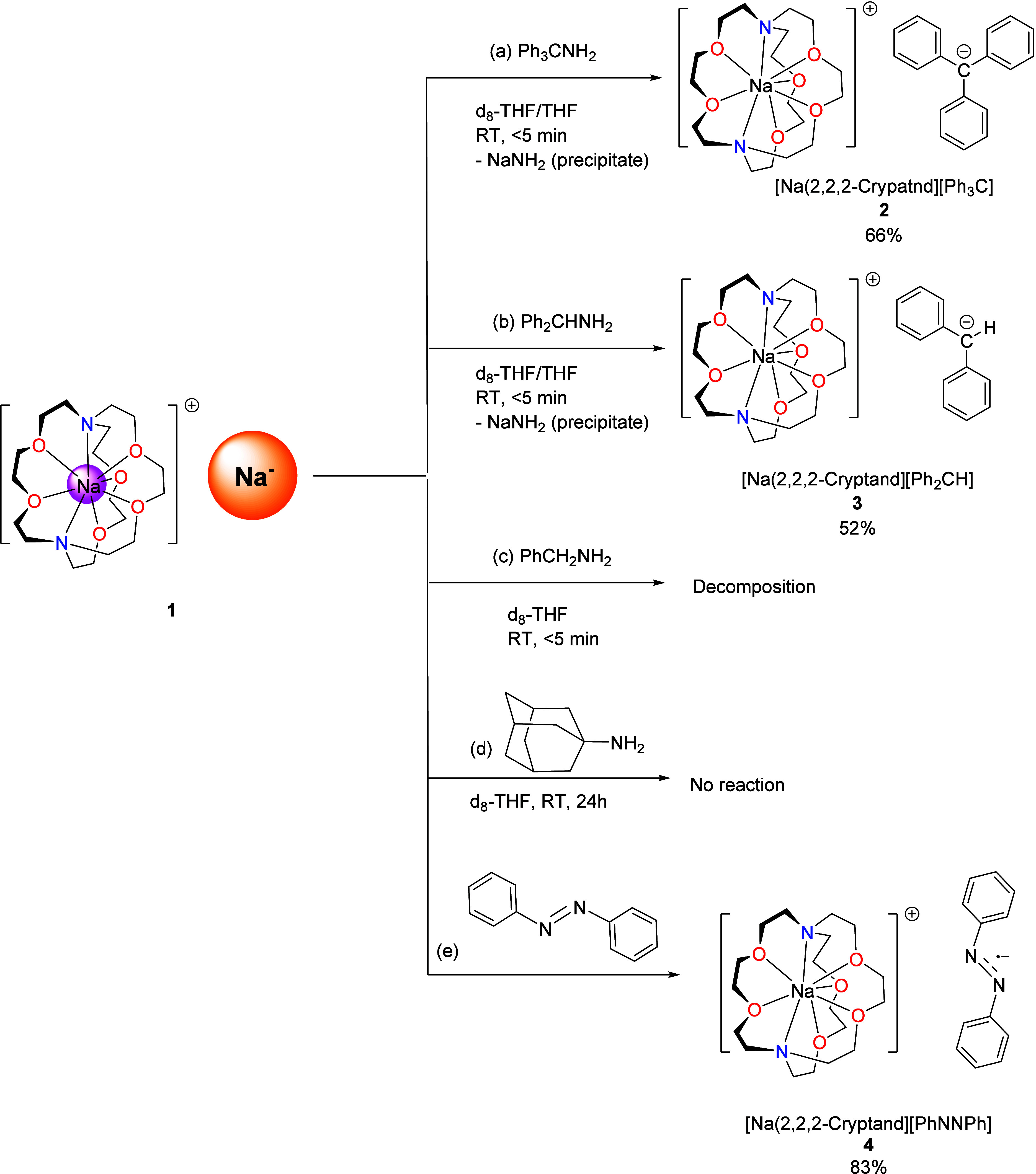
Overview of the reactivity of **1** toward (a) Ph_3_CNH_2_, (b) Ph_2_CHNH_2_, (c) PhCH_2_NH_2_, (d) adamantylamine, and (e) azobenzene.

The reaction between **1** and 1 equiv of trityl amine (Ph_3_CNH_2_) in *d*_8_-THF (Figure 6a) resulted in an immediate color change from blue to red. ^1^H NMR monitoring of the reaction mixture indicated the formation of a clean product. Scaling up the reaction in THF and crystallization led to the isolation of red crystals, the structure of which was unveiled by the SCXRD study to be [Na(2,2,2-cryptand)]^+^[Ph_3_C]^−^ separated ion pair (SIP) complex **2** (Figure 6a). The SCXRD structure of **2** is shown in Figure S23. In the Ph_3_C^–^ anion, the central carbon atom features a distorted trigonal planar geometry with three C^cent^–C^ipso^ bonds with lengths of 1.44–1.47 Å. These structural parameters match those of the reported alkali metal trityl complexes.^47,48^

**Figure 7:**
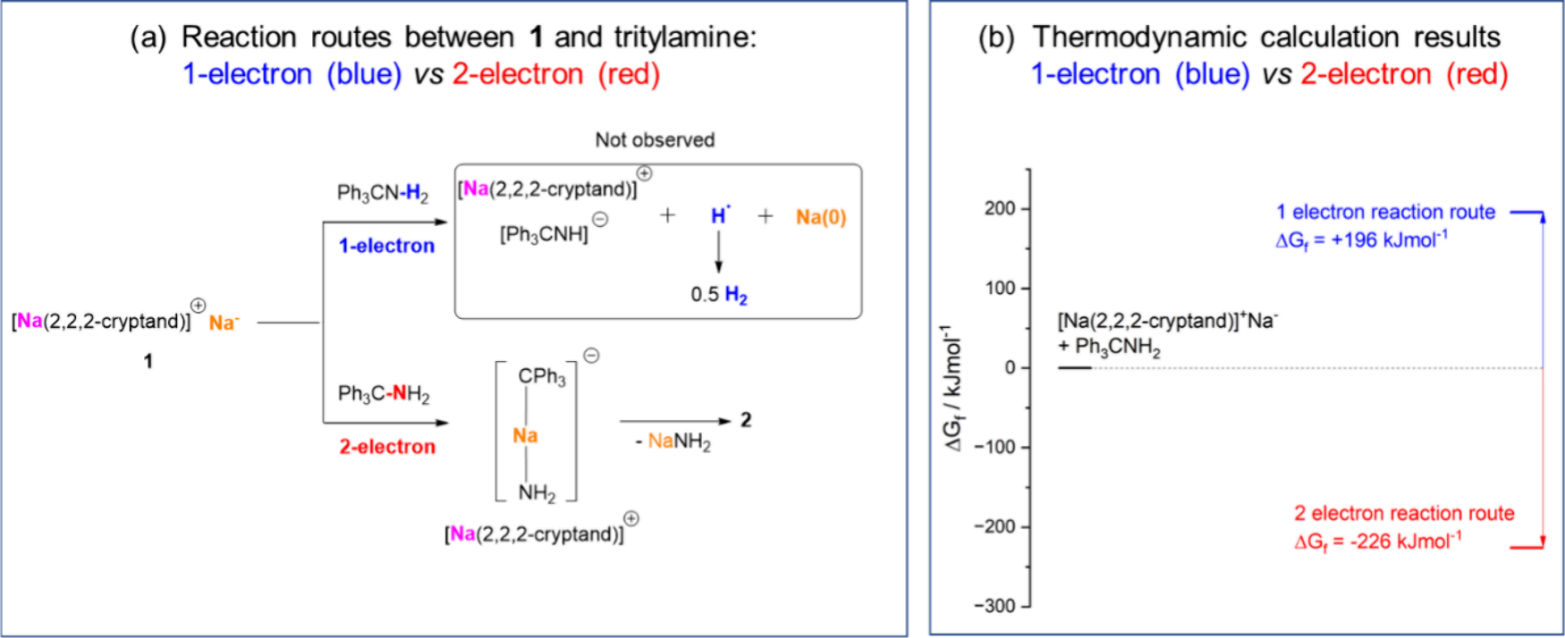
(a) Two possible reaction routes between **1** and trityl amine: one-electron reductive deprotonation vs two-electron oxidative addition/deamination (color codes indicate the different origins of Na in **1**, pink for Na^+^ and yellow for Na^–^). (b) Thermodynamic calculations for the two routes.

Given the challenging nature of primary amines’ C–N bond cleavage,^41,42^ the formation of **2** in high yield and under mild conditions is impressive. We rationalize **2** as the result of a two-electron oxidative addition of the Na^–^ center to the C–N bond in trityl amine, followed by the formation of NaNH_2_ and **2** (Figure 7a). The formation of NaNH_2_ could function as a thermodynamic driving force for the reaction. We did not observe any products from the potential one-electron reductive deprotonation, e.g., H_2_ or Na(0) species, or the two-electron oxidative addition toward the N–H bond. Hence, in the case of trityl amine, the Na^–^ center in **1** exhibits highly selective single-metal two-electron C–N bond cleavage and deamination. Thermodynamic calculations were conducted to understand the driving force of the selectivity (Figure 7b), with computational details given in Section 2 of the Supporting Information. The two-electron process was found to be exergonic, whereas the one-electron process was found to be endergonic; the differences in their Gibbs free energies of formation (Δ*G*_f_) were calculated to be −226 kJ/mol (two-electron) and 196 kJ/mol (one-electron). While this approach neglects a variety of different important factors such as kinetics and the entropic gains as a result of the evolution of a gaseous product (for the hypothetical one-electron reductive deprotonation), the exer/endergonic nature of the two reactions qualitatively agrees with the experimental findings, suggesting that the two-electron route is more thermodynamically favorable.

It is noteworthy that, despite the fact that the trityl group is a widely used protective group for amines,^49^ direct trityl amine deamination is scarce and requires a large excess of alkali metal on silica gel,^50^ or iodine (I_2_) to activate the -NH_2_.^51^ Similar to that of the trityl amine, the reaction between **1** and diphenylmethyl amine (Ph_2_CHNH_2_) produces corresponding deamination product [Na(2,2,2-cryptand)][Ph_2_CH] (**3**) (Figure 6b). The SCXRD structure of **3** is shown in Figure S24. It is noteworthy that some pale-colored precipitate was observed in both reactions to form **2** and **3**, which we postulated to be the NaNH_2_ byproduct. It is well-known that NaNH_2_ is insoluble in most hydrocarbon and ethereal solvents. Further reducing the steric bulkiness of the primary amine to benzyl amine (PhCH_2_NH_2_) leads to a fast reaction but decomposition (Figure 6c). We hypothesize that putative benzyl sodium SIP complex [Na(2,2,2-cryptand)][PhCH_2_] (which is unknown to the best of our knowledge^52^) is unstable and induces decomposition.

Interestingly, in sharp contrast with the fast reactions between **1** and Ph_3_CNH_2_, Ph_2_CHNH_2_, or PhCH_2_NH_2_, **1** does not react with 1-adamantylamine (Figure 6d) even after several hours at room temperature. There are two possible reasons for the inertness of 1-adamantylamine. (1) The adamantyl group^53^ is not as easy to reduce to the corresponding carbon anion as trityl or diphenylmethyl.^54^ (2) The steric rigidity of the adamantyl group is less favorable for the two-electron oxidative addition intermediate. It is also noteworthy that one-electron reductive deprotonation was not observed in any of the three primary amines tested; i.e., the N–H bond did not react.

**Figure 8:**
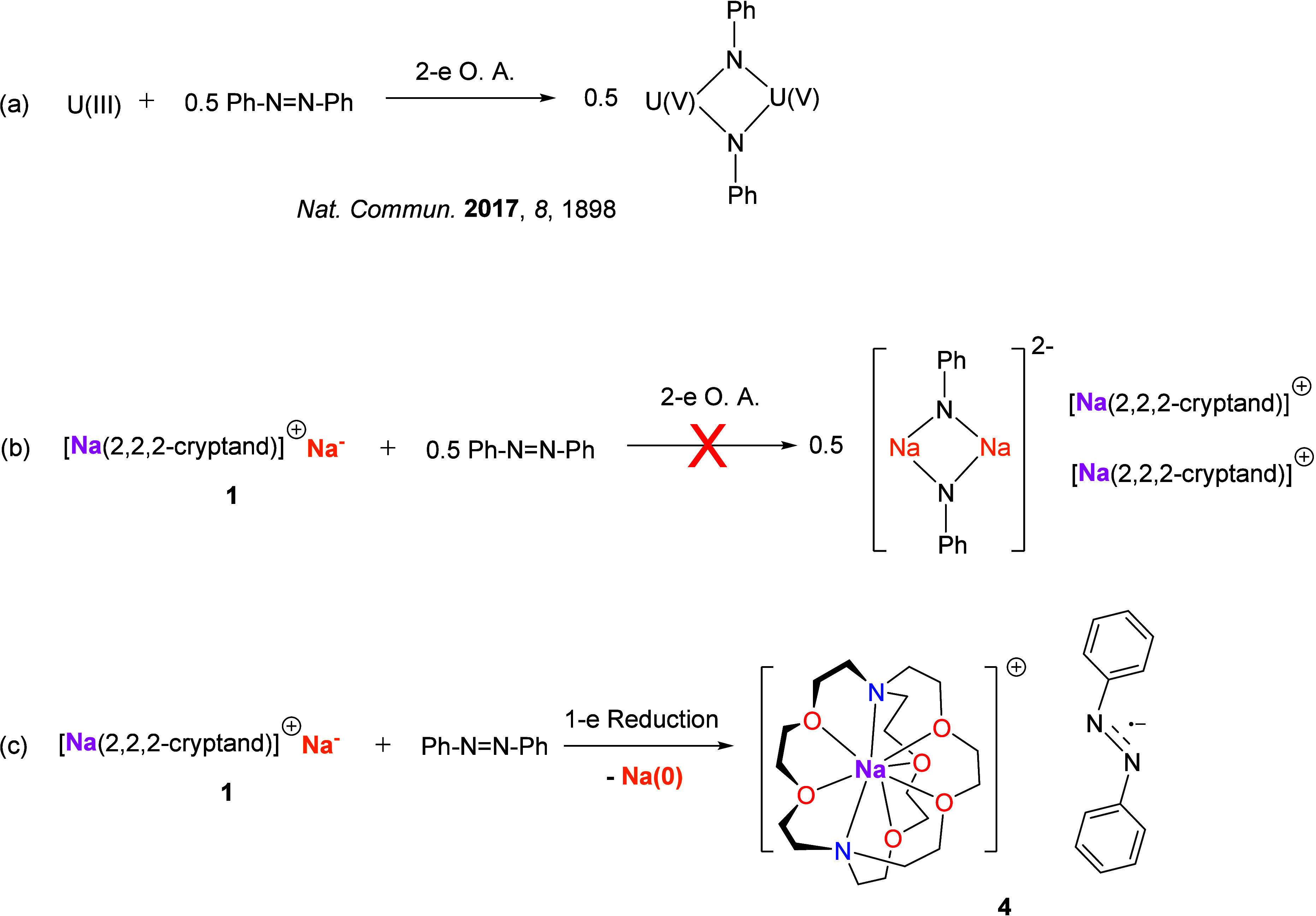
(a) Uranium-mediated two-electron oxidative addition toward azobenzene.^44^ (b) Hypothetical two-electron oxidative addition between **1** and azobenzene, mimicking the uranium pattern, which was not observed herein. (c) What actually happened: one-electron reduction of azobenzene to produce SIP complex **3**, featuring an azobenzene radical monoanion.

It is now obvious that in the cases of primary amines, **1** prefers the two-electron process over the one-electron process. Nevertheless, the scenario is different for azobenzene. We tested the reaction between **1** and 0.5 equiv of azobenzene (Figure 8b), aiming to mimic the reported U(+3) ↔ U(+5) two-electron oxidative addition (Figure 8a).^44^ However, ^1^H and ^23^Na NMR monitoring of the reaction in *d*_8_-THF indicates the presence of unreacted **1**, along with new broad ^1^H NMR signals, which could be a new paramagnetic product(s). Hence, we increased the amount of azobenzene and increased the stoichiometric ratio to 1:1 (Figure 8c). Immediately after mixing at room temperature, the 1:1 reaction in *d*_8_-THF produced a black/purple solution with a significant amount of gray/black fine solid precipitate, where the *in situ*^1^H and ^23^Na NMR spectra indicate the full consumption of both of the starting materials, and exhibited a Na^+ 23^Na signal and a ^1^H NMR spectrum with featureless and wide signals. Scaling up the 1:1 reaction in THF and recrystallization from a THF/hexane mixture under −35 °C produced purple crystals, which were confirmed by SCXRD to be an azobenzene radical monoanion SIP complex [Na(2,2,2-cryptand)][PhNNPh] (**4**) (Figures 6e and 8c). The SCXRD structure of **4** is shown in Figure S25, where the bond lengths and angles in the azobenzene radical monoanion match with the reported precedents.^55^ The ^1^H NMR spectrum of **4** is featureless in the aromatic region, which corroborates its radical nature, as reflected in the X-band solution EPR study (Figure S21). The radical property is further consolidated by computational studies (*vide infra*).

We conducted a DFT molecular orbital analysis of **4**. The singly occupied molecular orbital (SOMO) is delocalized across the [PhNNPh]^•–^ radical anion. In corresponding with the delocalized SOMO, spin density analysis indicated that the spin density is delocalized, as well. Such spin density delocalization has been widely reported in main-group radical complexes.^56^ For the MO and spin density analyses of **4**, see the Supporting Information. We postulate that the Na(0) species, as the byproduct of the one-electron reduction, precipitate out as the gray/black solid and hence cannot be observed in the *in situ* solution-state ^23^Na NMR spectrum in *d*_8_-THF.

## Conclusion

In conclusion, in this work, the first facile synthesis of sodide **1** allowed us to study systematically the sodide reactivity for the first time. We realized the first group 1 single-metal two-electron reduction in a challenging transformation, primary amine deamination. The two-electron versus one-electron reactivity of **1** is dependent on the substrate. **1** exhibits one-electron reduction toward azobenzene. Further work is underway in our group in two directions: (1) expanding the easy-access alkalide scope and (2) exploring the alkalides’ potential in inert small molecule and chemical bond activations, particularly focusing on exploiting the new AM(−1) ↔ AM(+1) redox couple to mimic two-electron transition metal catalysis.

## Experimental Section

### General Procedures

All manipulations were carried out in a Vigor glovebox equipped with a −35 °C freezer and a cold well, under an atmosphere of dry argon. Solvents were dried with sodium press, a sodium–potassium alloy, distilled under reduced pressure, and kept in the glovebox. Chemicals were purchased from Merck, Fluorochem, or Alfa Aesar and dried under dynamic vacuum for several hours (for solids) or with activated 4 Å molecular sieves that had been frozen, thawed, and vacuum degassed (for liquid) prior to use. All glassware, including pipettes, vials, and ampules, was silylated by being treated with trimethylsilyl chloride (Me_3_SiCl), rinsed with water, and dried in a 150 °C oven for 12 h prior to use.

Mechanochemical ball milling reactions were conducted using a Retsch MM400 mixer mill within a 25 mL Retsch stainless steel jar and a 10 mm diameter stainless steel ball (one ball per jar). For the *in situ* visual monitoring study, a 20 mm diameter three-dimensionally printed PMMA jar was used, which was sealed with electric tape in the glovebox.

Solution-state ^1^H, ^23^Na, and ^13^C{^1^H} NMR spectra were recorded on a Bruker 400 Avance III spectrometer or a Bruker 500 Avance III HD spectrometer operating at 400 or 500 MHz, respectively, for ^1^H NMR, 132 or 106 MHz, respectively, for ^23^Na NMR, and 101 or 126 MHz, respectively, for ^13^C{^1^H} NMR. Solid-state MAS ^23^Na NMR was recorded on a Bruker 500 Avance III HD NMR spectrometer, operating at 132 MHz, with 4 mm rotors and a MAS rate of 10 kHz. Chemical shifts are quoted in parts per million and are relative to SiMe_4_ (^1^H and ^13^C{^1^H}) or external 0.1 M NaCl in D_2_O (solution-state ^23^Na) or external solid NaCl (solid-state MAS ^23^Na).

Continuous-wave (CW) electron paramagnetic resonance (EPR) spectra were recorded on a Bruker EMX EPR spectrometer operating at the X-band frequency (9.4 GHz).

### Synthesis and Characterization of [Na^+^(2,2,2-cryptand)Na^–^] (**1**)

On a 1 mmol scale, [2.2.2]cryptand (0.3765 g, 1 mmol) and sodium metal (0.0460 g, 2 mmol) were combined in a 25 mL ball milling jar, with a 10 mm stainless steel ball. The jar was sealed in an argon glovebox. The mixture was ball milled on a Retch MM400 ball mill at 30 Hz for 10 min. During ball milling, the jar turned slightly warm. The jars were opened in the glovebox. A bronze powder resulted.

For the SCXRD study, the bronze powder was dissolved in THF (12 mL), and the resulting deep blue solution was filtered and placed in a −35 °C freezer. After 24 h, gold crystals suitable for SCXRD resulted. The mother liquor was removed, and the volatiles were removed *in vacuo*. A gold crystalline powder resulted (0.1612 g, 38% yield).

On a 2 mmol scale, [2.2.2]cryptand (0.7530 g, 2 mmol) and sodium metal (0.0920 g, 4 mmol) were combined in a 25 mL ball milling jar, with a 10 mm stainless steel ball. The jar was sealed in an argon glovebox. The mixture was ball milled on a Retch MM400 ball mill at 30 Hz for 10 min. During ball milling, the jar turned slightly warm. The jars were opened in the glovebox. A bronze powder resulted.

For crystallization, the bronze powder was dissolved in THF (10 mL), and the resulting deep blue solution was filtered and placed in a −35 °C freezer. After 24 h, gold crystals resulted. The mother liquor was removed, and the volatiles were removed *in vacuo*. A gold crystalline powder resulted (0.4023 g, 48% yield): ^1^H NMR (500 MHz, *d*_8_-THF, 25 °C) δ 3.63 (s, br, 12H, C*H*_2_), 3.58 (s, br, 12H, C*H*_2_), 2.64 (s, br, 12H, C*H*_2_); ^23^Na NMR (106 MHz, *d*_8_-THF, 25 °C) δ 1132.2 (Na^0^), −11.0 (Na^+^), −62.1 (Na^–^); ^23^Na NMR (132 MHz, MAS solid-state, 25 °C) δ −10.3 (Na^+^), −61.1 (Na^–^).

### Reactivity of [Na^+^(2,2,2-cryptand)Na^–^] (**1**)

#### Reaction of [Na^+^(2,2,2-cryptand)Na^–^] (**1**) with Triphenylmethylamine to Produce **2**

On a NMR scale, **1** (0.0169 g, 0.04 mmol) was dissolved in *d*_8_-THF (0.25 mL). The resulting dark blue solution was added in one portion to a colorless solution of triphenylmethylamine (0.0104 g, 0.04 mmol) in *d*_8_-THF (0.25 mL) that had been cooled in a −35 °C freezer. A deep red solution resulted. The solution was transferred to a J. Young NMR tube, and the reaction was monitored by ^1^H and ^23^Na NMR.

For scaling up, **1** (0.1098 g, 0.26 mmol) was dissolved in THF (1.5 mL). The resulting dark blue solution was added in one portion to a colorless solution of triphenylmethylamine (0.0674 g, 0.26 mmol) in THF (1 mL) that had been cooled in a −35 °C freezer. A deep red solution resulted. The solution was left at room temperature for 45 min, before being filtered. *n*-Hexane (0.5 mL) was added, and the solution was placed in a −35 °C freezer. Crystals suitable for SCXRD resulted after 30 min. The mother liquor was removed, and the crystals were washed with *n*-hexane (2 mL) and dried *in vacuo*. Red crystals resulted (0.1100 g, 66% yield): ^1^H NMR (400 MHz, *d*_8_-THF, 25 °C) δ 7.29 (d, *J* = 8.2 Hz, Ar*H*, 6H), 6.50 (t, *J* = 7.7 Hz, Ar*H*, 6H), 5.97–5.88 (m, Ar*H*, 3H), 3.54 (s, C*H*_2_, 12H), 3.52–3.47 (m, C*H*_2_, 12H), 2.61–2.54 (m, C*H*_2_, 12H); ^23^Na NMR (106 MHz, *d*_8_-THF, 25 °C) δ −11.1 (Na^+^); ^13^C{^1^H} NMR (101 MHz, *d*_8_-THF, 25 °C) δ 150.1, 128.1, 124.4, 112.9, 69.4, 68.5, 53.8.

#### Reaction of [Na^+^(2,2,2-cryptand)Na^–^] (**1**) with Benzhydrylamine to Produce **3**

On a NMR scale, **1** (0.0169 g, 0.04 mmol) was dissolved in *d*_8_-THF (0.25 mL). The resulting dark blue solution was added in one portion to a colorless solution of benzhydrylamine (0.0073 g, 0.04 mmol) in *d*_8_-THF (0.25 mL) that had been cooled in a −35 °C freezer. A dark red/orange cloudy solution resulted. The solution was transferred to a J. Young NMR tube, and the reaction was monitored by ^1^H and ^23^Na NMR.

For scaling up, **1** (0.1056 g, 0.25 mmol) was dissolved in THF (1.5 mL). The resulting dark blue solution was added in one portion to a colorless solution of benzhydrylamine (0.0458 g, 0.25 mmol) in THF (1.5 mL) that had been cooled in a −35 °C freezer. A deep red/orange cloudy solution resulted. The solution was left at room temperature for 30 min, before being filtered twice, and the solution was placed in a −35 °C freezer. Crystals suitable for SCXRD resulted overnight. The mother liquor was removed, and the crystals were washed with *n*-hexane (2 × 1 mL) and dried *in vacuo*. Red crystals resulted (0.0738 g, 52% yield): ^1^H NMR (400 MHz, *d*_8_-THF, 25 °C) δ 6.66–6.25 (m, Ar*H*, 8H), 5.61–5.46 (m, Ar*H*, 2H), 4.32 (s, C*H*, 1H), 3.44 (s, C*H*_2_, 12H), 3.41 (t, *J* = 5.0 Hz, C*H*_2_, 12H), 2.47 (t, *J* = 4.8 Hz, C*H*_2_, 12H); ^23^Na NMR (106 MHz, *d*_8_-THF, 25 °C) δ −10.8 (Na^+^); ^13^C{^1^H} NMR (101 MHz, *d*_8_-THF, 25 °C) δ 147.1, 128.1, 106.0, 82.0, 69.3, 68.5, 53.8.

#### Reaction of [Na^+^(2,2,2-cryptand)Na^–^] (**1**) with Benzylamine

On a NMR scale, **1** (0.0169 g, 0.04 mmol) was dissolved in *d*_8_-THF (0.25 mL). The resulting dark blue solution was added in one portion to a colorless solution of benzylamine (0.0043 g, 0.04 mmol) in *d*_8_-THF (0.25 mL) that had been cooled in a −35 °C freezer. A dark blue solution resulted, which was transferred to a J. Young NMR tube. Within 5 min, the solution turned into a cloudy pale blue/gray mixture with insolubles, which is suspected to be due to fast decomposition of the reaction product. ^1^H and ^23^Na NMR was recorded (Figures S17 and S18).

#### Reaction of [Na^+^(2,2,2-cryptand)Na^–^] (**1**) with Azobenzene to Produce 4

On a NMR scale, **1** (0.0169 g, 0.04 mmol) was dissolved in *d*_8_-THF (0.25 mL). The resulting dark blue solution was added in one portion to a colorless solution of azobenzene (0.0073 g, 0.04 mmol) in *d*_8_-THF (0.25 mL) that had been cooled in a −35 °C freezer. A cloudy dark red/brown solution resulted. The solution was transferred to a J. Young NMR tube, and the reaction was monitored by ^1^H and ^23^Na NMR.

For scaling up, **1** (0.0670 g, 0.16 mmol) was dissolved in THF (1.5 mL). The resulting dark blue solution was added in one portion to an orange solution of azobenzene (0.0292 g, 0.16 mmol) in THF (1.5 mL) that had been cooled in a −35 °C freezer. A brown cloudy solution resulted. The solution was left at room temperature for 30 min before the volatiles were removed *in vacuo*, and the crystalline solid was washed with *n*-hexane (2 × 2 mL) and dried *in vacuo* (0.0770 g, 83% yield).

Purple crystals suitable for SCXRD resulted after 6 h from a THF (5 mL) solution that was filtered, followed by the addition of *n*-hexane (1 mL), and placed in a −35 °C freezer: ^1^H NMR (400 MHz, *d*_8_-THF, 25 °C) δ 3.35 (s, br, C*H*_2_, 24H), 2.47 (s, br, C*H*_2_, 12H) (the [PhNNPh]^•–^ fragment is NMR silent); ^23^Na NMR (106 MHz, *d*_8_-THF, 25 °C) δ −10.5 (Na^+^).

#### Attempted Reaction of [Na^+^(2,2,2-cryptand)Na^–^] (**1**) with 1-Adamantylamine

On a NMR scale, **1** (0.0169 g, 0.04 mmol) was dissolved in *d*_8_-THF (0.5 mL). The resulting dark blue solution was added in one portion to a colorless solution of 1-adamantylamine (0.0061 g, 0.04 mmol) in *d*_8_-THF (0.5 mL) that had been cooled in a −35 °C freezer. No observable color change resulted. The solution was transferred to a J. Young NMR tube, and the reaction was monitored by ^1^H and ^23^Na NMR, which showed no reaction between **1** and 1-adamantylamine and decomposition of **1** within 1 day.

## Supporting Information Available

Additional experimental details, materials, and methods, including photographs of the experimental setup (PDF)

## Dedication

This paper is in memory of Professor Li-Xin Dai, Shanghai Institute of Organic Chemistry, who sadly passed away on May 13, 2024 (1924–2024).
