# Supplementary material for: Mechanochemical Synthesis of a Sodium Anion Complex [Na+(2,2,2-cryptand)Na–] and Studies of Its Reactivity: Two-Electron and One-Electron Reductions
Source: Inorg Chem. 2024 Jul 29;63(32):15247–58. doi: 10.1021/acs.inorgchem.4c02914 (PMC11323275; doi:10.1021/acs.inorgchem.4c02914)
Supplement: Supplementary file 1 — ic4c02914_si_001.pdf [file ic4c02914_si_001.pdf]

Supplementary Materials for

Mechanochemical synthesis of a sodium anion complex [Na^+(2,2,2-cryptand)Na^-] and its reactivity studies: 2-electron and 1-electron reductions

Nathan Davison [a,b], Jack M. Hemingway [b] *, Corinne Wills [b] *, Tomislav Stolar [c], Paul G. Waddell [b] *, Casey M. Dixon [b], Luke Barron [b], James A. Dawson [b], and Erli Lu [a,b] *§

Affiliations:

[a] School of Chemistry, University of Birmingham, Edgbaston, Birmingham, B15 2TT (UK)

[b] Chemistry–School of Natural and Environmental Sciences, Newcastle University, Newcastle upon Tyne, NE1 7RU (UK)

[c] Federal Institute for Materials Research and Testing (BAM), 12489 Berlin (Germany)

*Corresponding authors:
Erli.Lu@newcastle.ac.uk (E. L.: The lead author)
jack.hemingway@newcastle.ac.uk (J. M. H.: Computations)
paul.waddell@newcastle.ac.uk (P. G. W.: Crystallography)
corinne.wills@newcastle.ac.uk (C. W.: Spectroscopies)

Homepage: www.erlilulab.org

X: https://twitter.com/erli_lu

§ The lead author.

This PDF file includes:

Materials and Methods
Supplementary Text
Figures S1 to S27
Tables S1 to S6

Section 1. Experimental methods and data---S2
Section 2. Computational details and data---S26
References--S31

Materials and Methods

Section 1. Experimental methods and data

1.1Spectroscopic characterizations of [Na^+(2,2,2-cryptand)Na^-] (**1**)

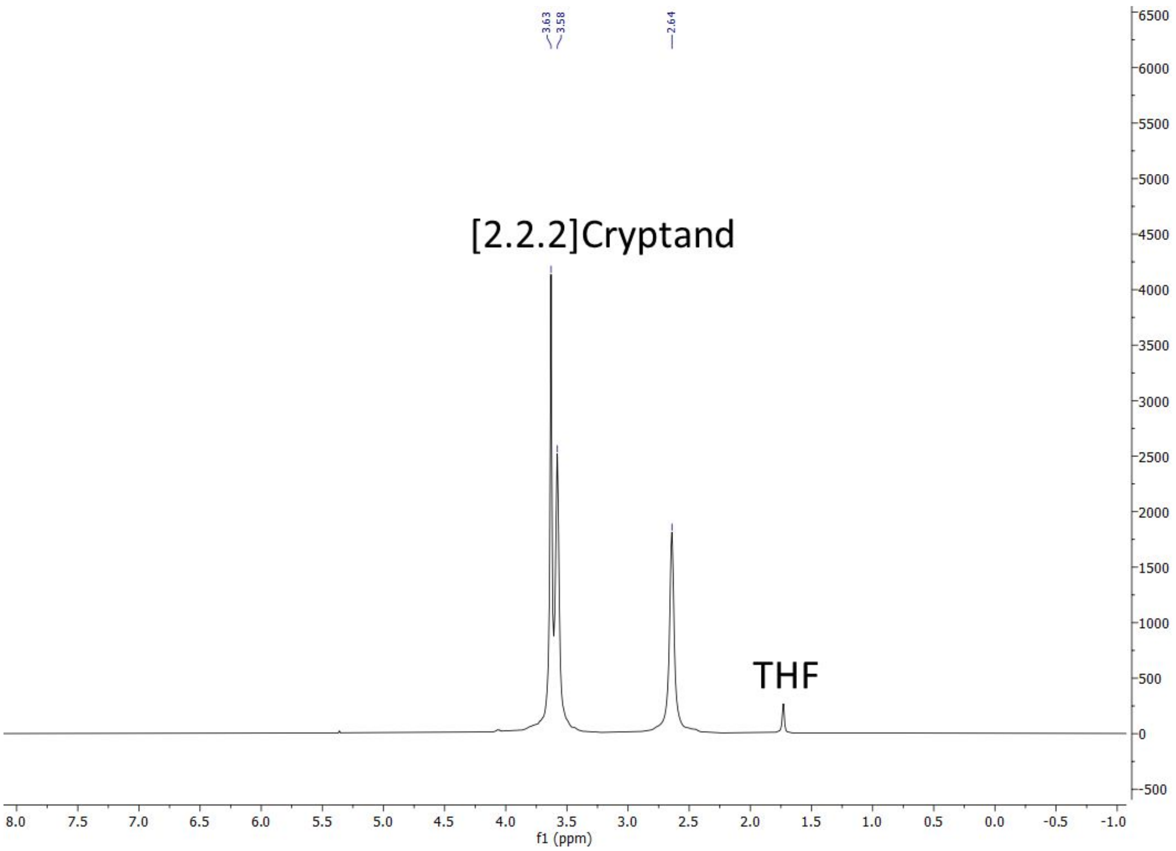

Figure S1: ^{1}H NMR (500 MHz, 25 °C) of crystalline [Na^+(2,2,2-cryptand)Na^-] (**1**) in d_8-THF solution.

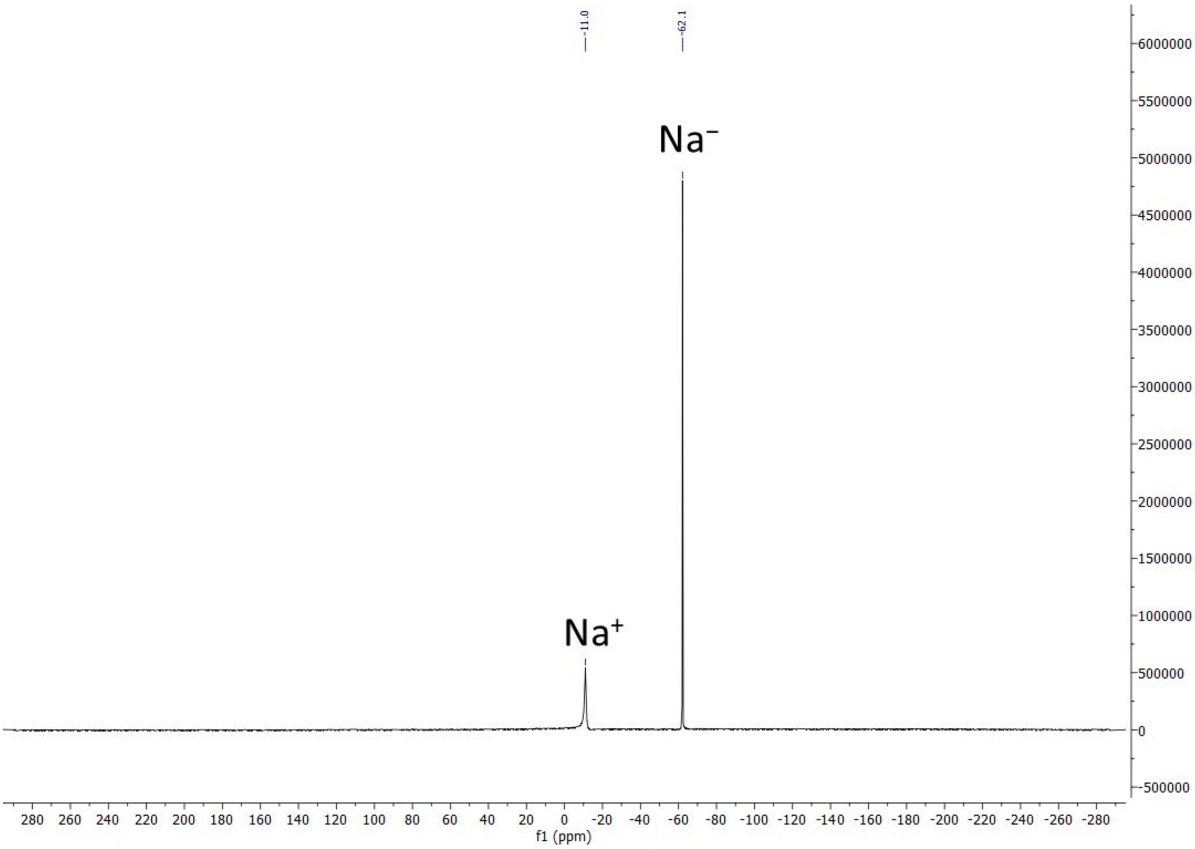

Figure S2: ^{23}Na NMR (106 MHz, 25 °C) of crystalline $[Na^+(2,2,2\text{-cryptand})Na^-]$ (**1**) in d_8-THF solution. O1P = 0 ppm.

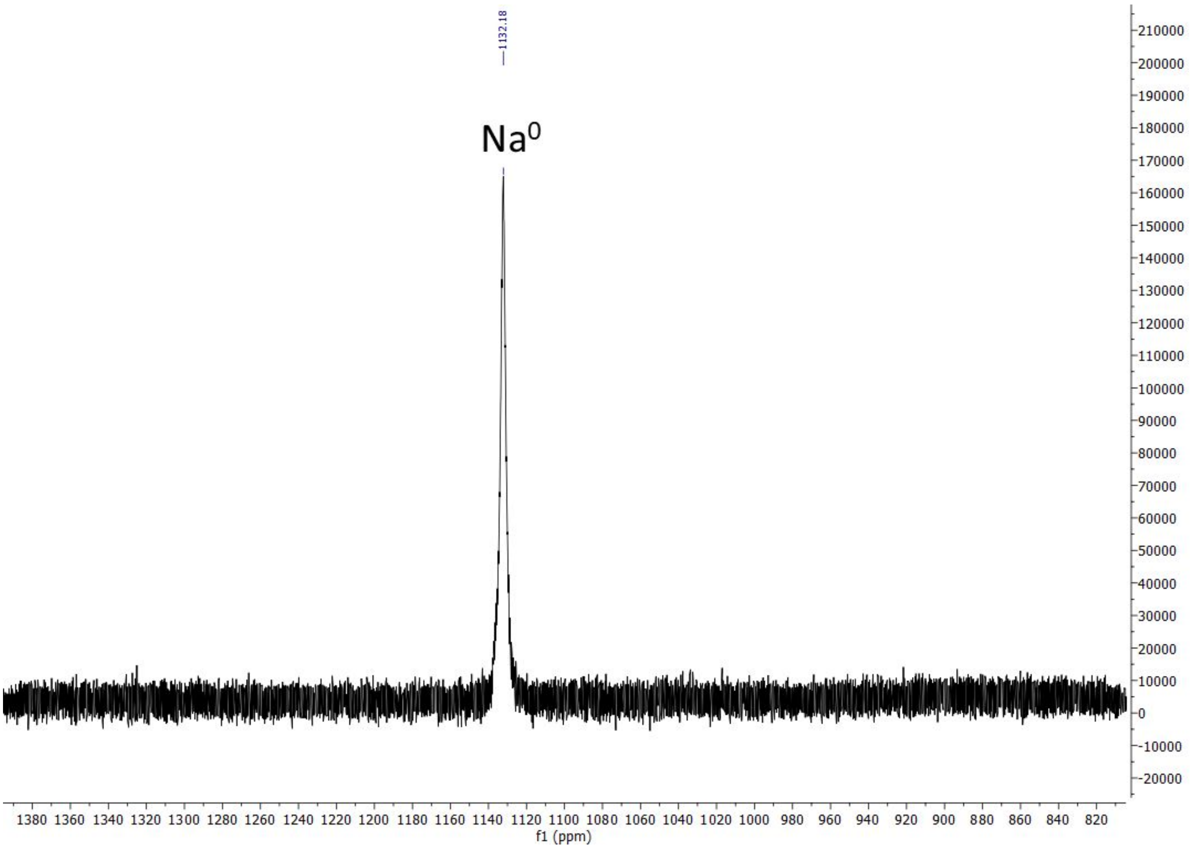

Figure S3: ^{23}Na NMR (106 MHz, 25 °C) of crystalline [Na^+(2,2,2-cryptand)Na^-] (**1**) in d_8-THF solution. OP1 = 1100 ppm.

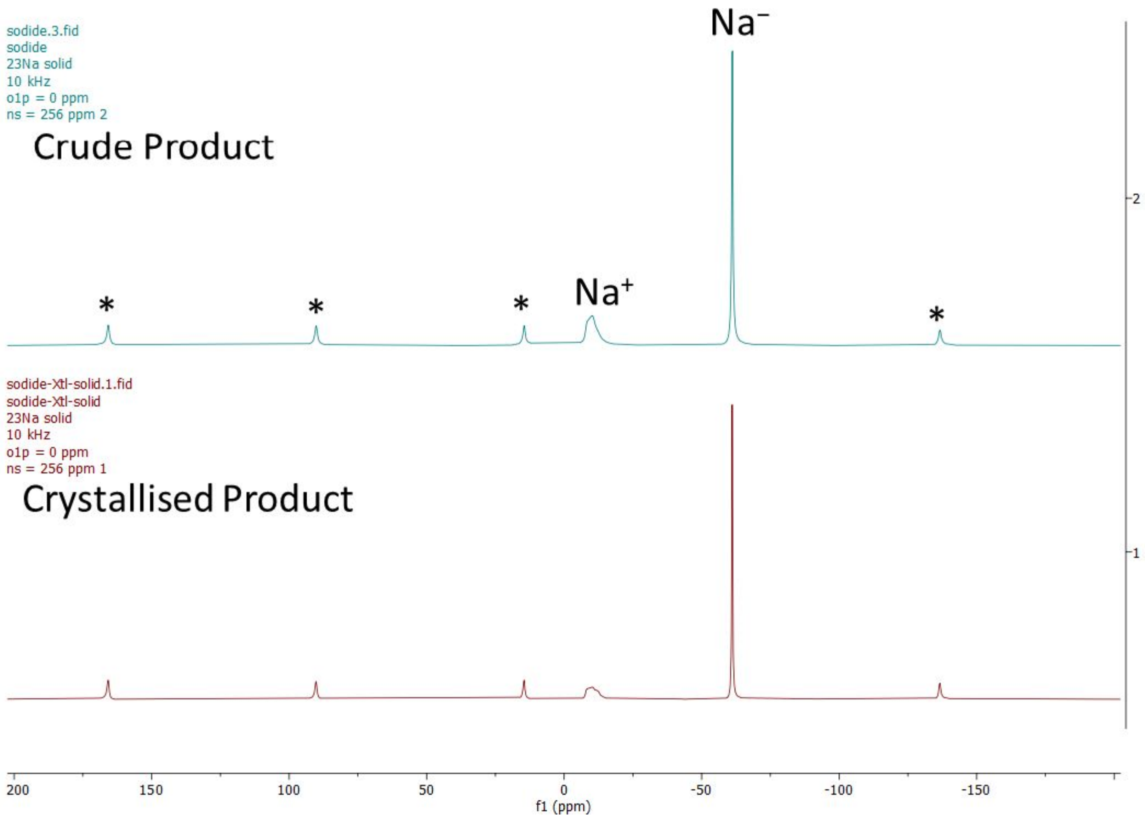

Figure S4: Stacked ^{23}Na (132 MHz, 25 °C) MAS solid-state NMR of [Na^+(2,2,2-cryptand)Na^-] (**1**). O1P = 0 ppm. Top (cyan): crude product. Bottom (blue): crystallised product. * = Spinning side bands.

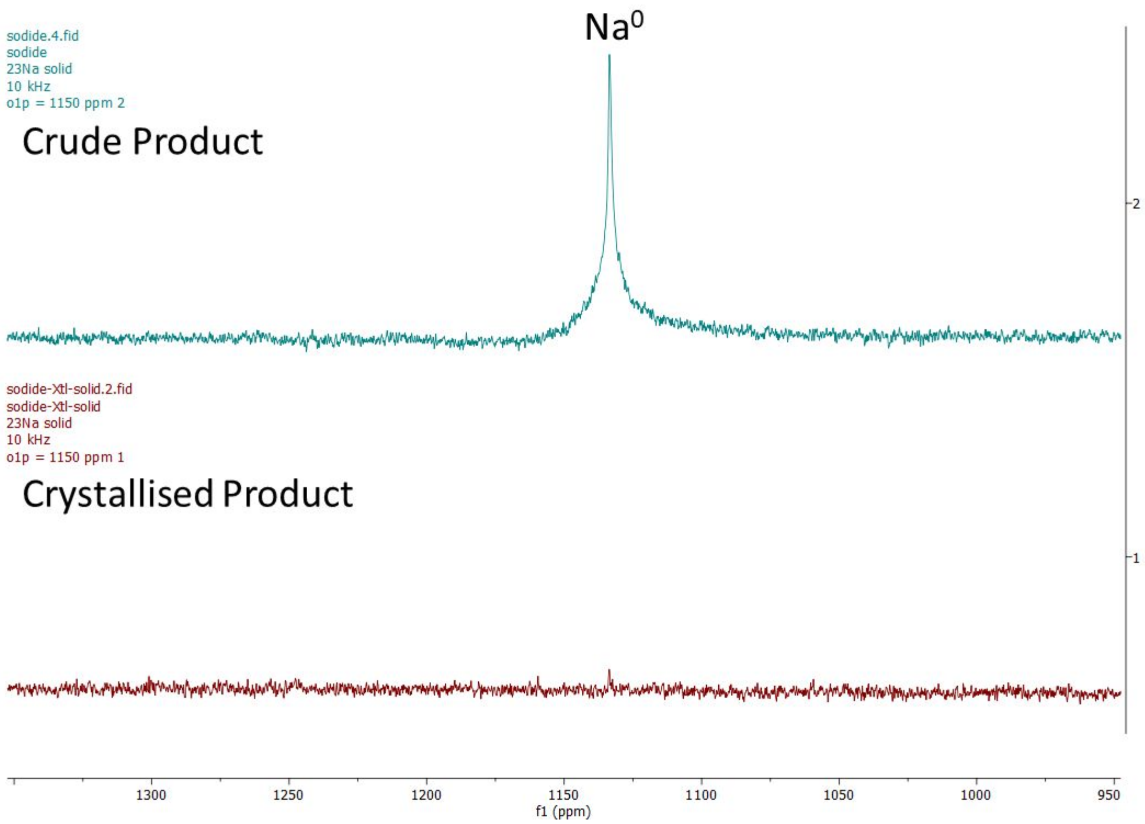

Figure S5: Stacked ^{23}Na (132 MHz, 25 °C) MAS solid-state NMR of [Na^+(2,2,2-cryptand)Na^-] (**1**). O1P = 1150 ppm. Top (cyan): crude product. Bottom (blue): crystallised product.

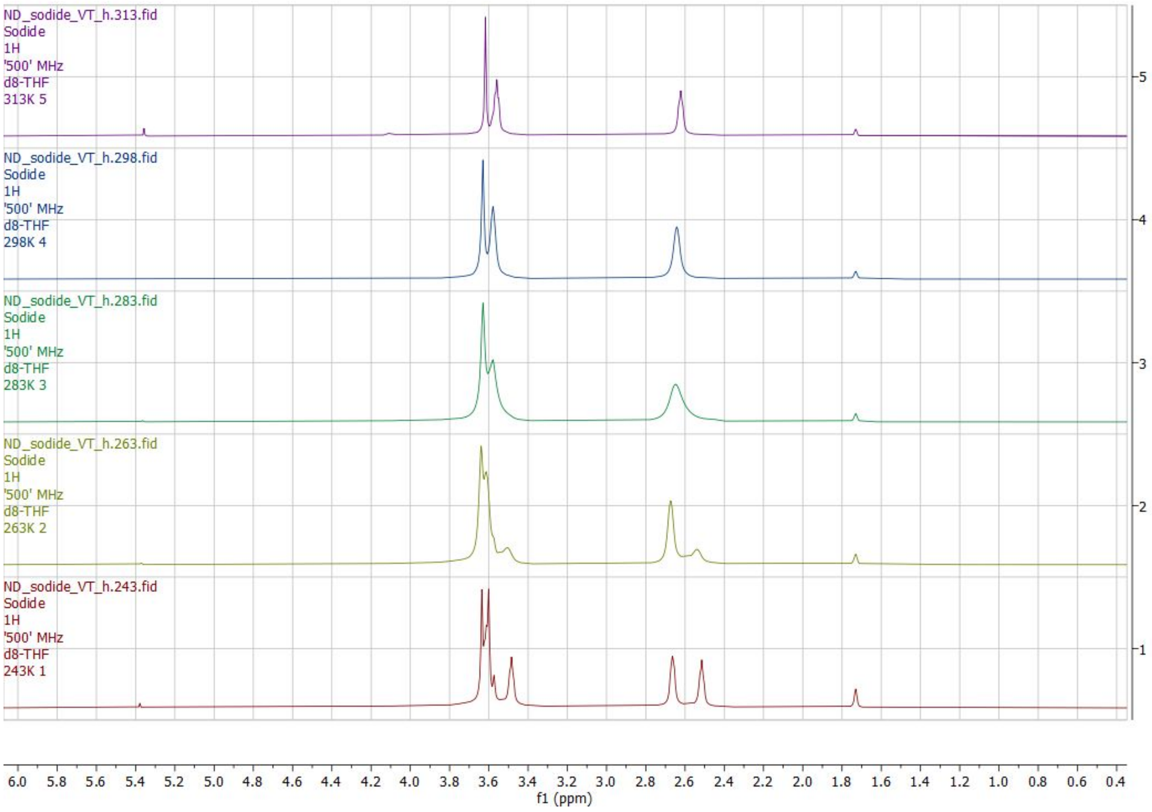

Figure S6: Stacked ^{1}H (500 MHz) variable temperature NMR on crystalline [Na^{+}(2,2,2-cryptand)Na^{-}] (**1**) in d_8-THF.

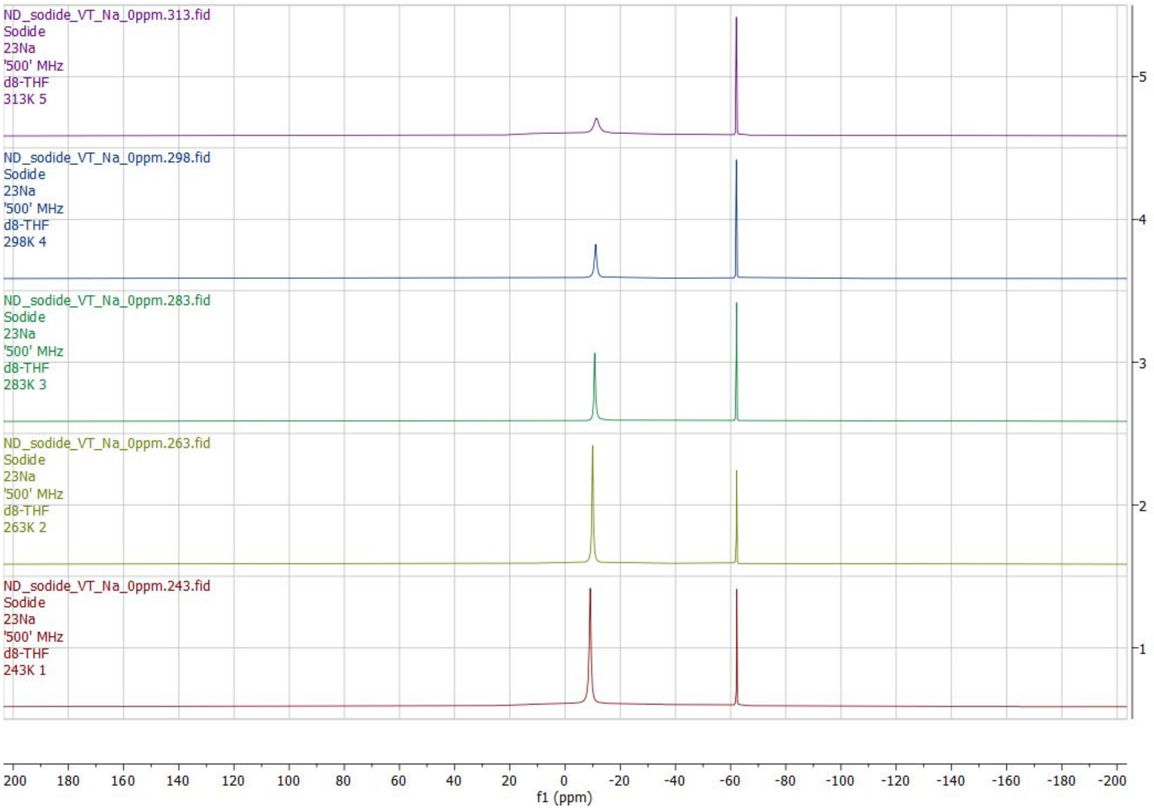

Figure S7: Stacked ^{23}Na (132 MHz) variable temperature NMR on crystalline [Na^{+}(2,2,2-cryptand)Na^{-}] (**1**) in d_8-THF. O1P = 0 ppm.

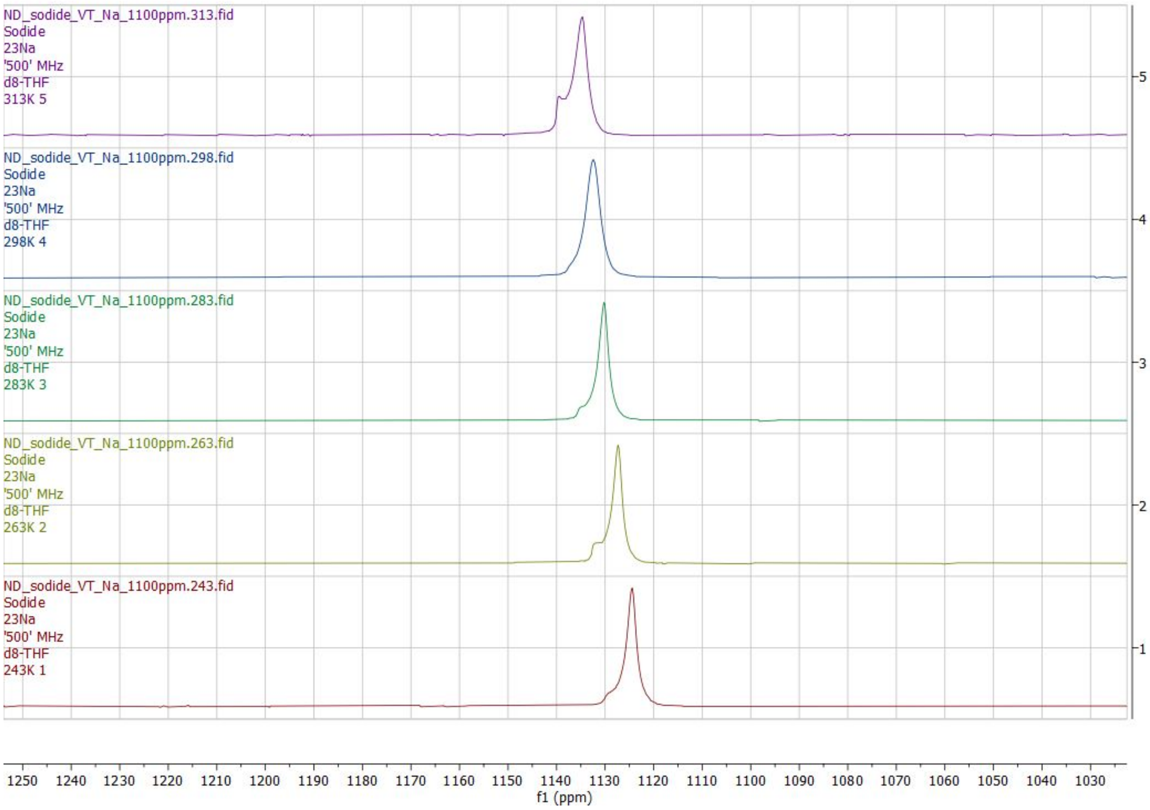

Figure S8: Stacked ^{23}Na (132 MHz) variable temperature NMR on crystalline [Na^{+}(2,2,2-cryptand)Na^{-}] (**1**) in d_8-THF. O1P = 1100 ppm.

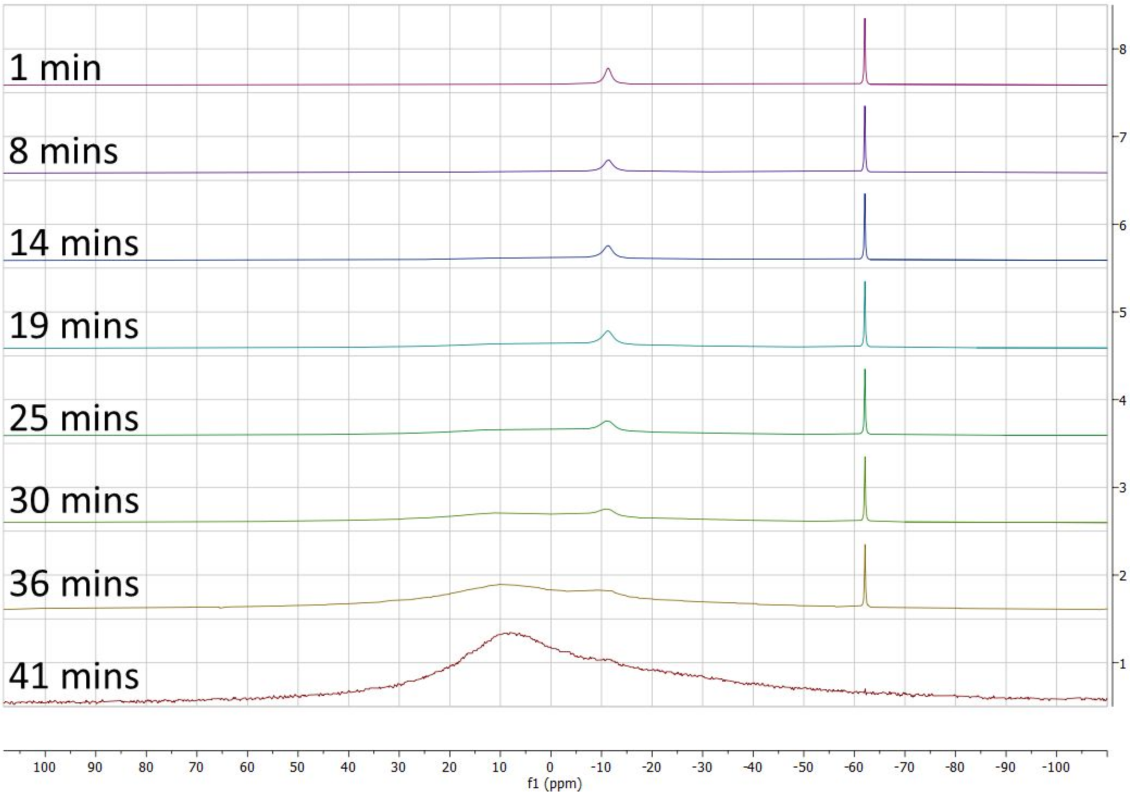

Figure S9: Stacked [23]Na (132 MHz, 313 K) decomposition study at 313K on crystalline [Na^+(2,2,2-cryptand)Na^-] (**1**) (0.0017 g) in d_8-THF (0.5 ml) with O1P at 0 ppm.

Time point = time since temperature reached 313 K. The total time the sample was at room temperature and the time taken to reach 313 K = 27 minutes prior to time point 0.

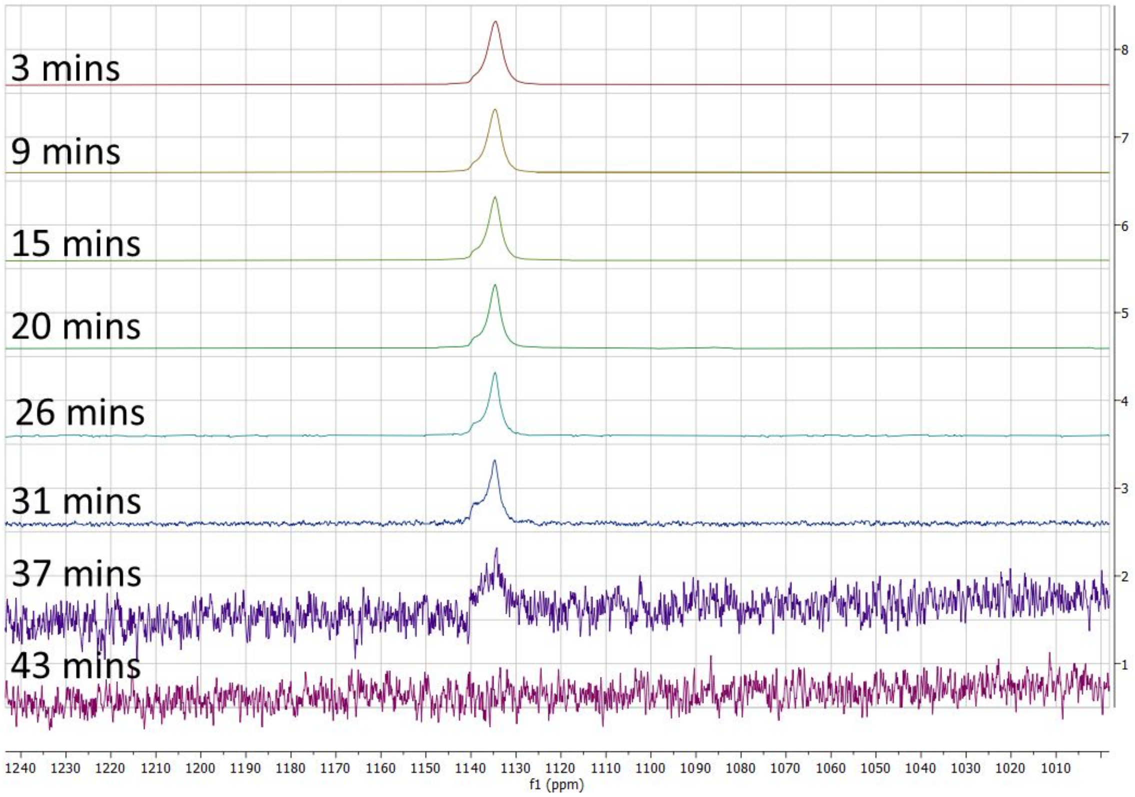

Figure S10: Stacked [23]Na (132 MHz, 313 K) decomposition study at 313K on crystalline [Na^+(2,2,2-cryptand)Na^-] (**1**) (0.0017 g) in d_8-THF (0.5 ml) with O1P at 1100 ppm.

Time point = time since temperature reached 313 K. The total time the sample was at room temperature and the time taken to reach 313 K = 27 minutes prior to time point 0.

1.2 NMR spectroscopies of **2**

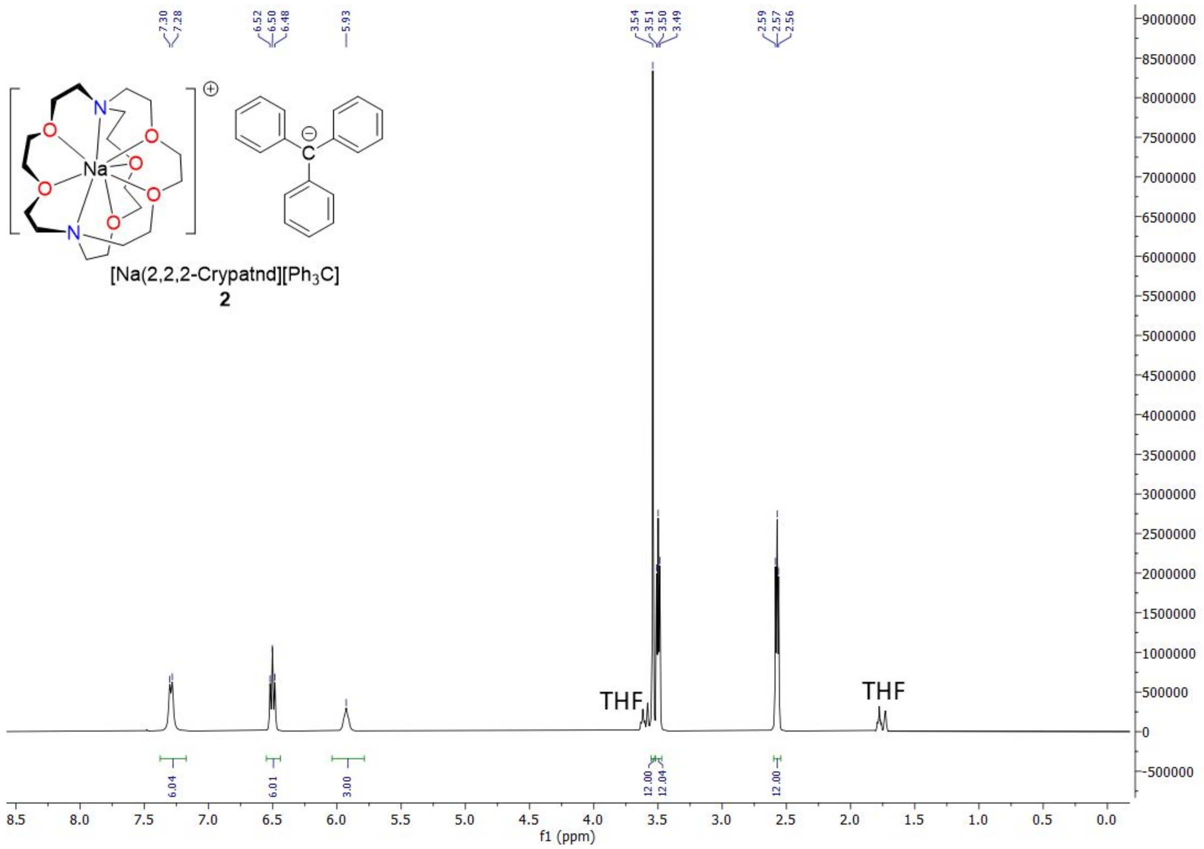

Figure S11: ^{1}H NMR (400 MHz, 25 °C) of crystalline **2** in d_8-THF.

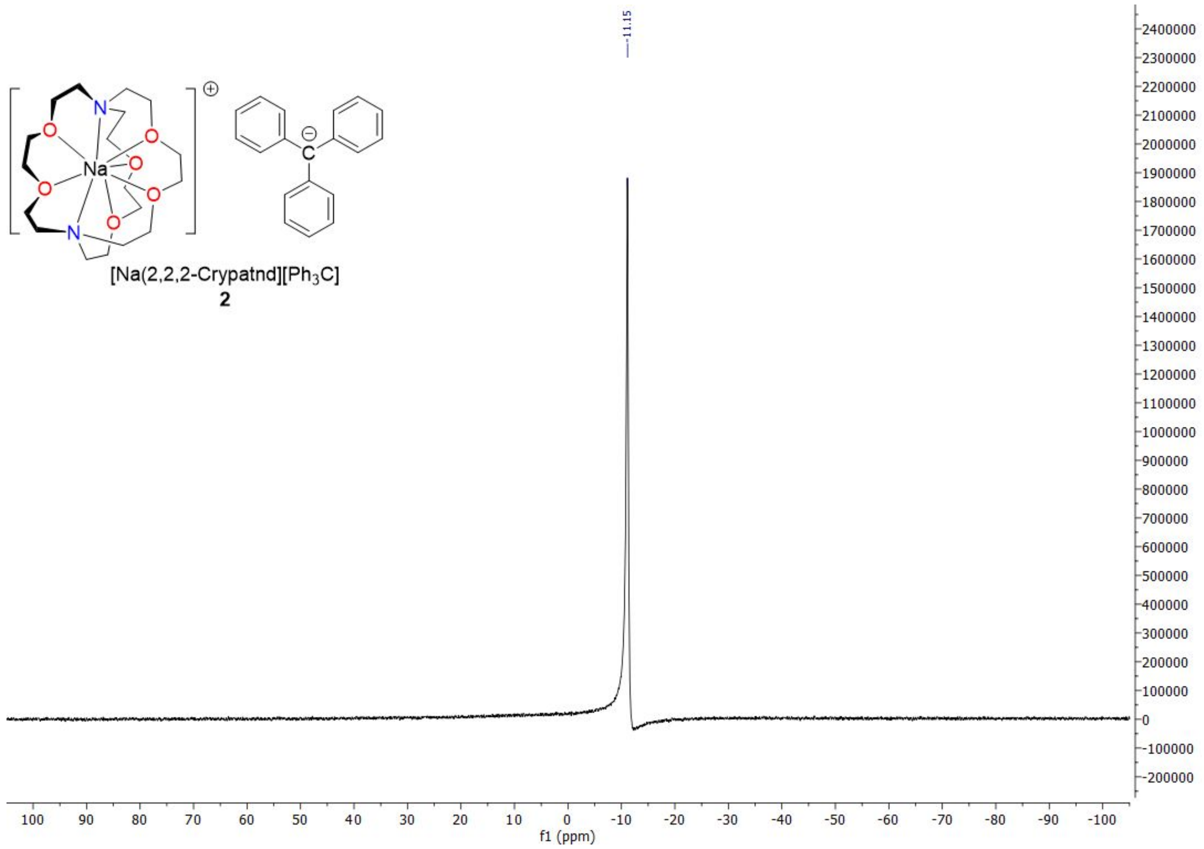

Figure S12: ^{23}Na NMR (106 MHz, 25 °C) of crystalline **2** in d_8-THF.

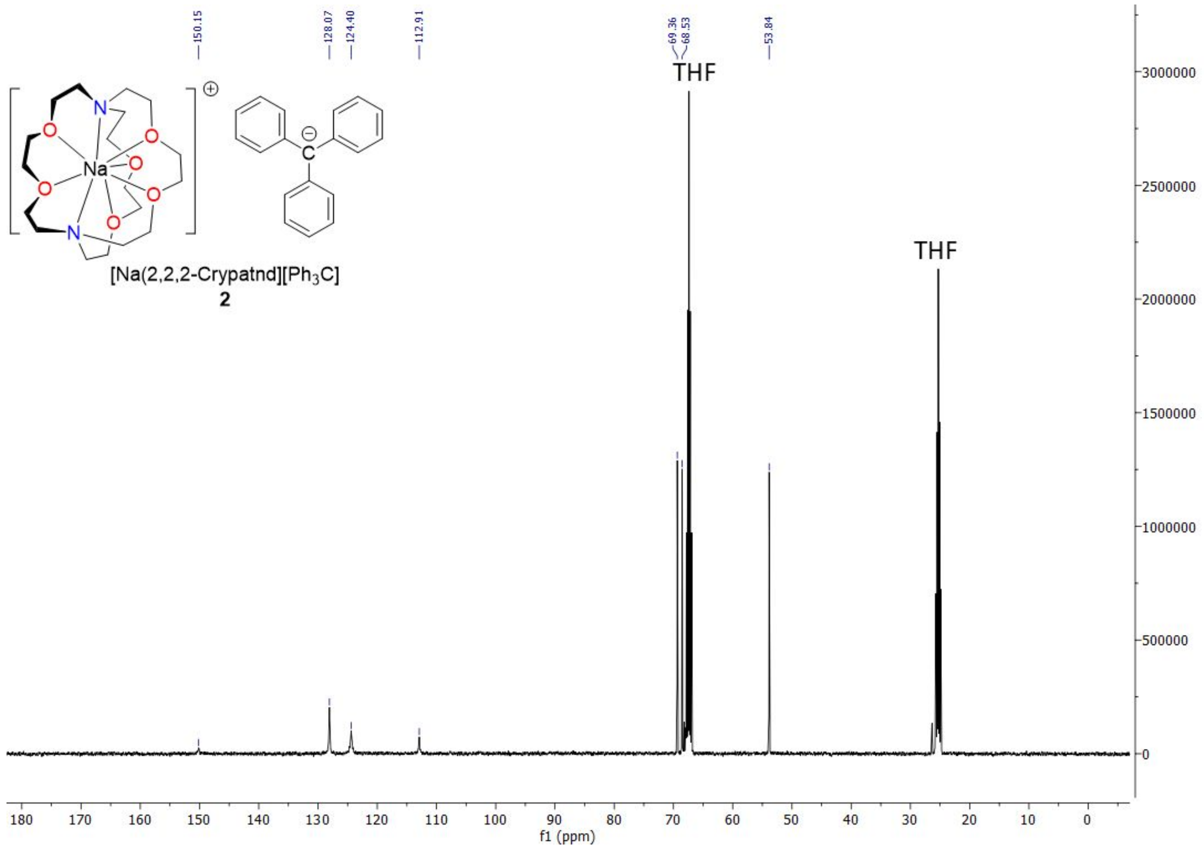

Figure S13: ^{13}C{^{1}H} NMR (101 MHz, 25 °C) of crystalline **2** in d_8-THF.

1.3 NMR spectroscopies of **3**

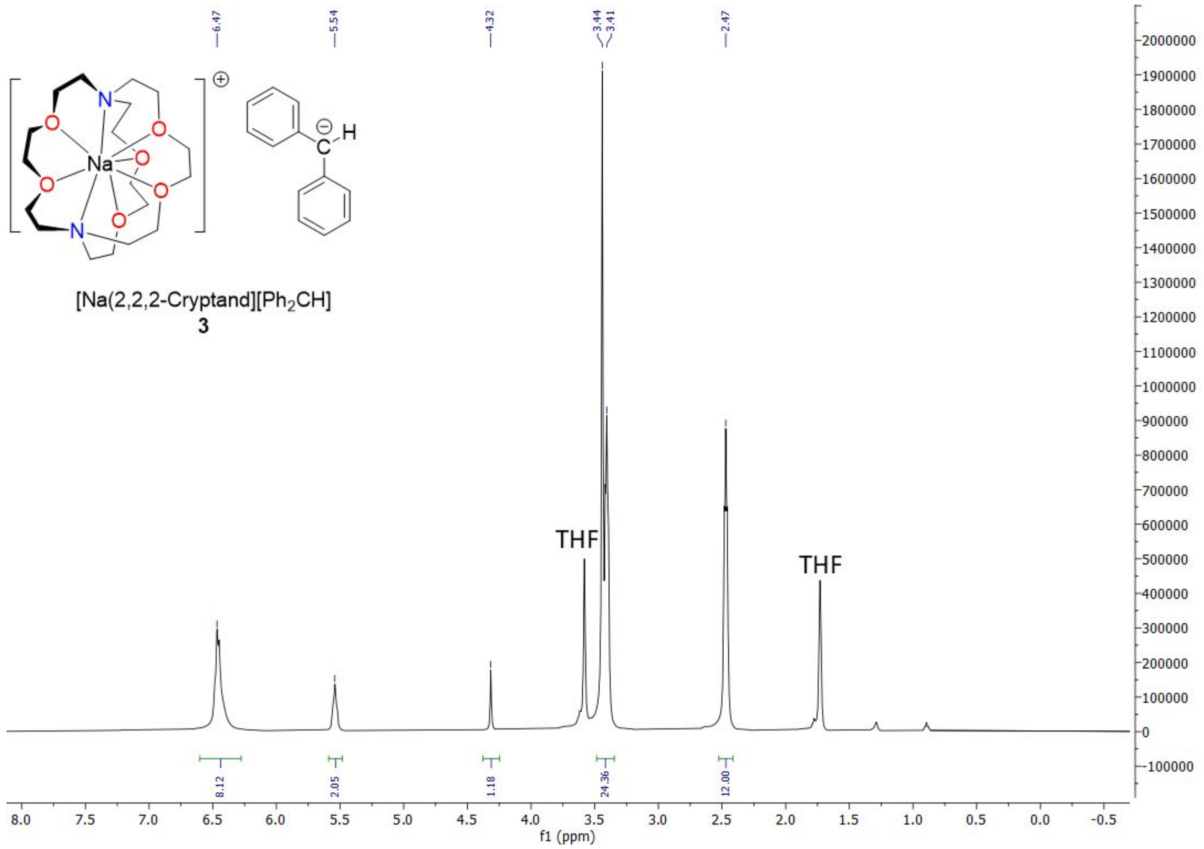

Figure S14: ^{1}H NMR (400 MHz, 25 °C) of crystalline **3** in d_8-THF.

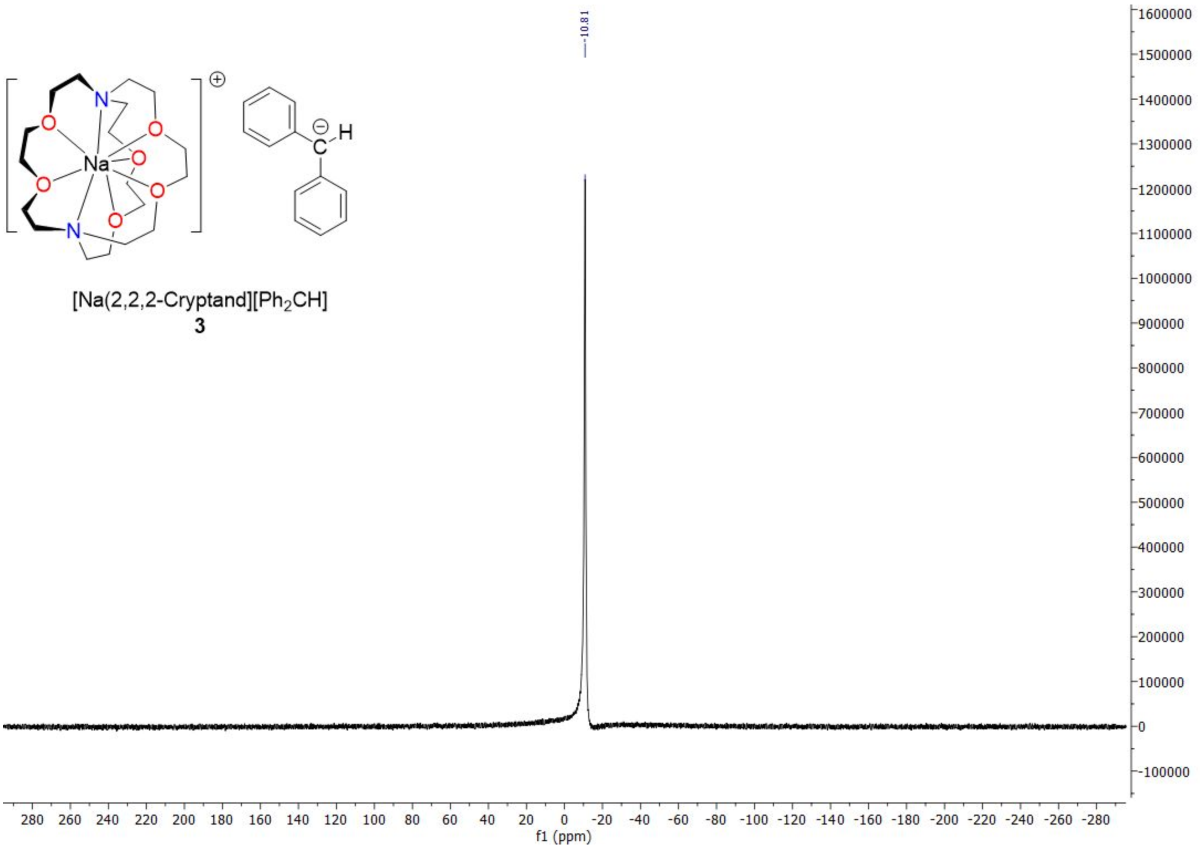

Figure S15: ^{23}Na NMR (106 MHz, 25 °C) of crystalline **3** in d_8-THF.

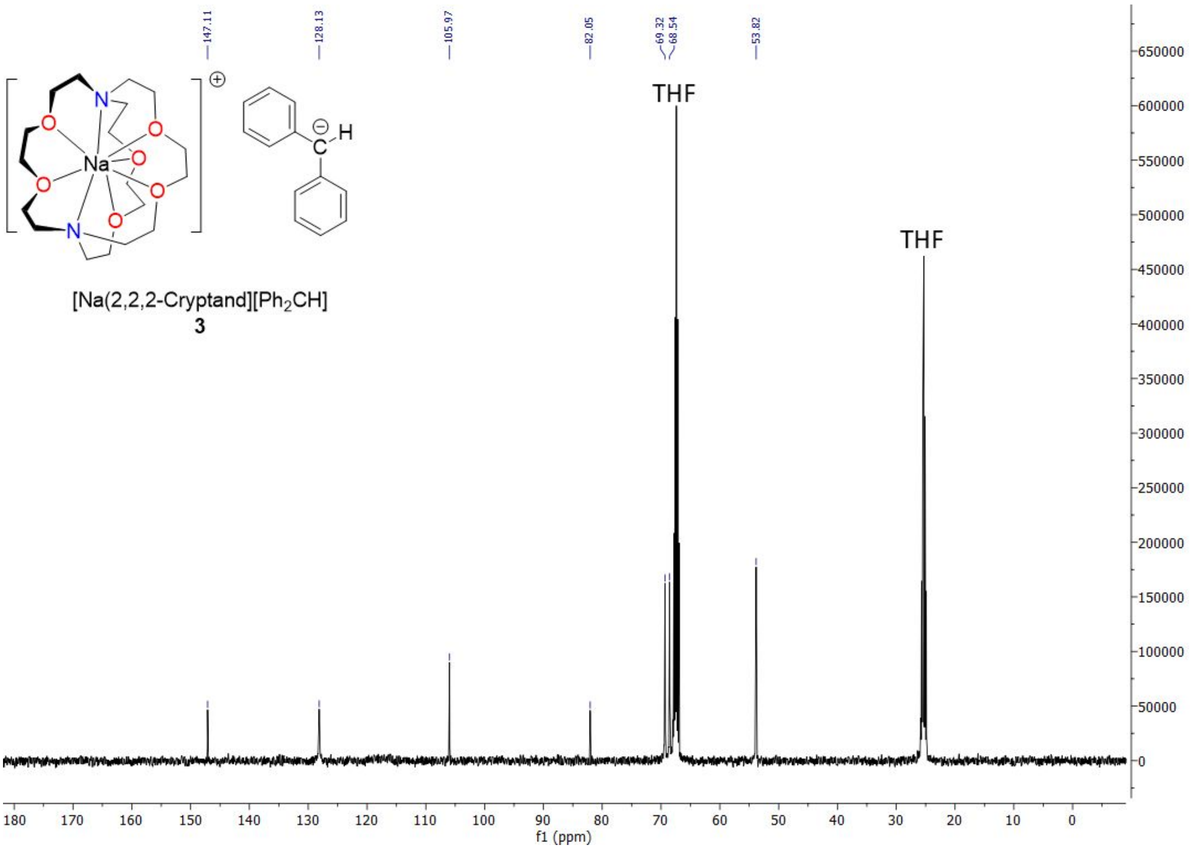

Figure S16: $^{13}C\{^{1}H\}$ NMR (101 MHz, 25 °C) of crystalline **3** in d_8-THF.

1.4 ^{1}H and ^{23}Na NMR spectra of the reaction between **1** and benzylamine

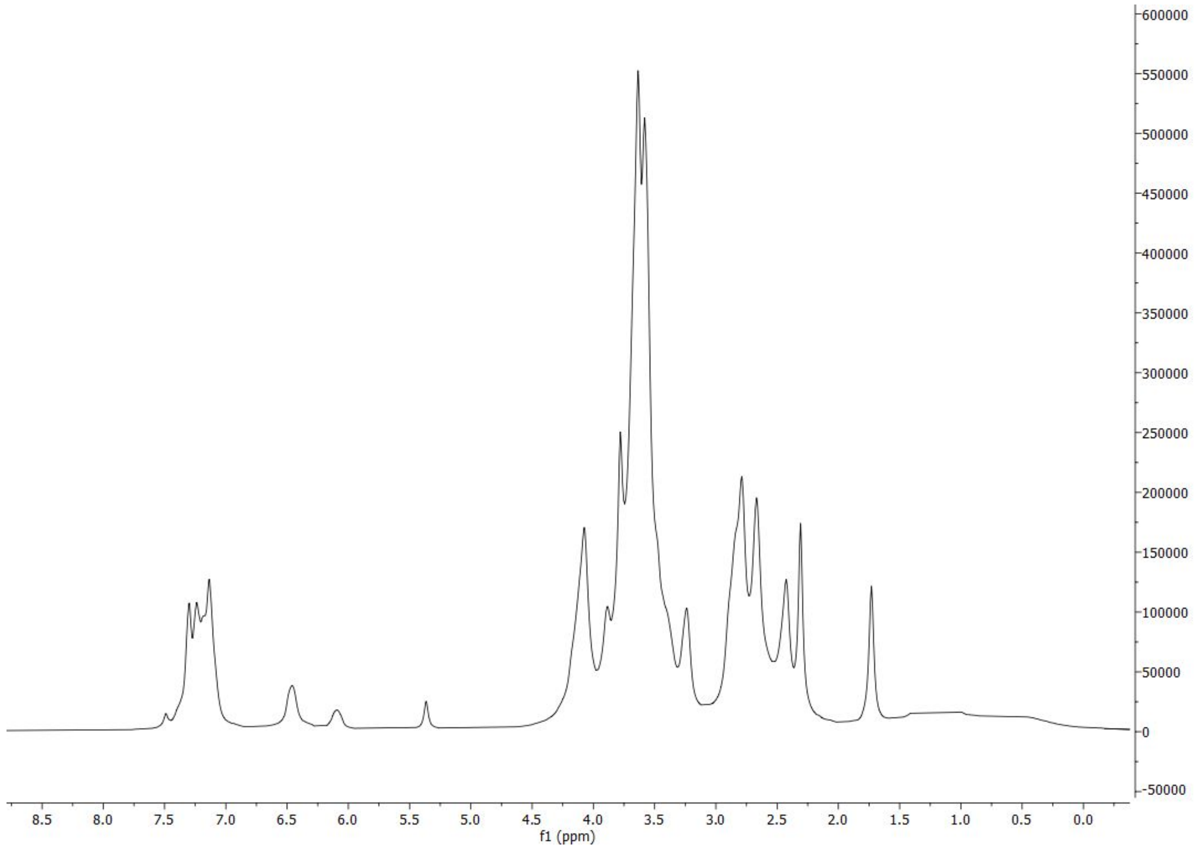

Figure S17: ^{1}H NMR (400 MHz, 25 °C) of an NMR scale reaction between **1** and benzylamine in d_8-THF.

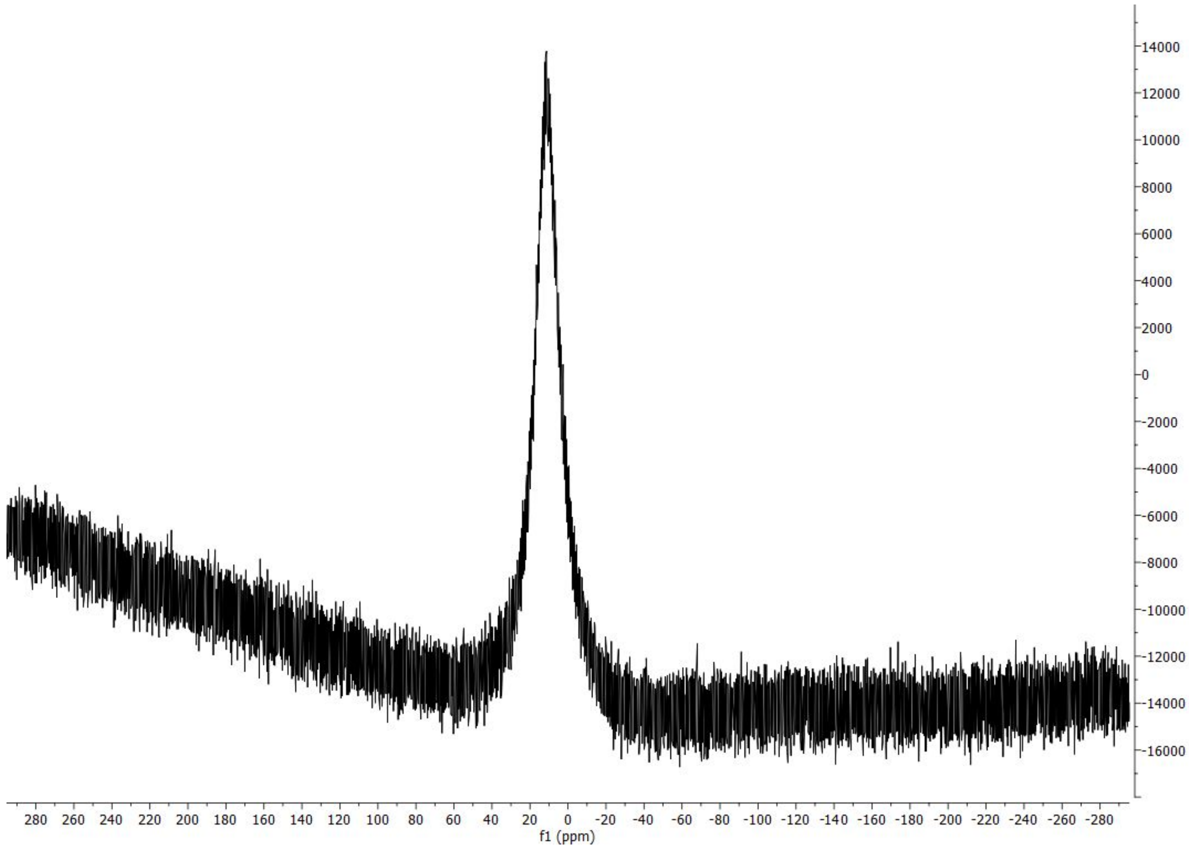

Figure S18: ^{23}Na NMR (106 MHz, 25 °C) of an NMR scale reaction between **1** and benzylamine in d_8-THF.

1.5 Spectroscopic characterisations of **4**

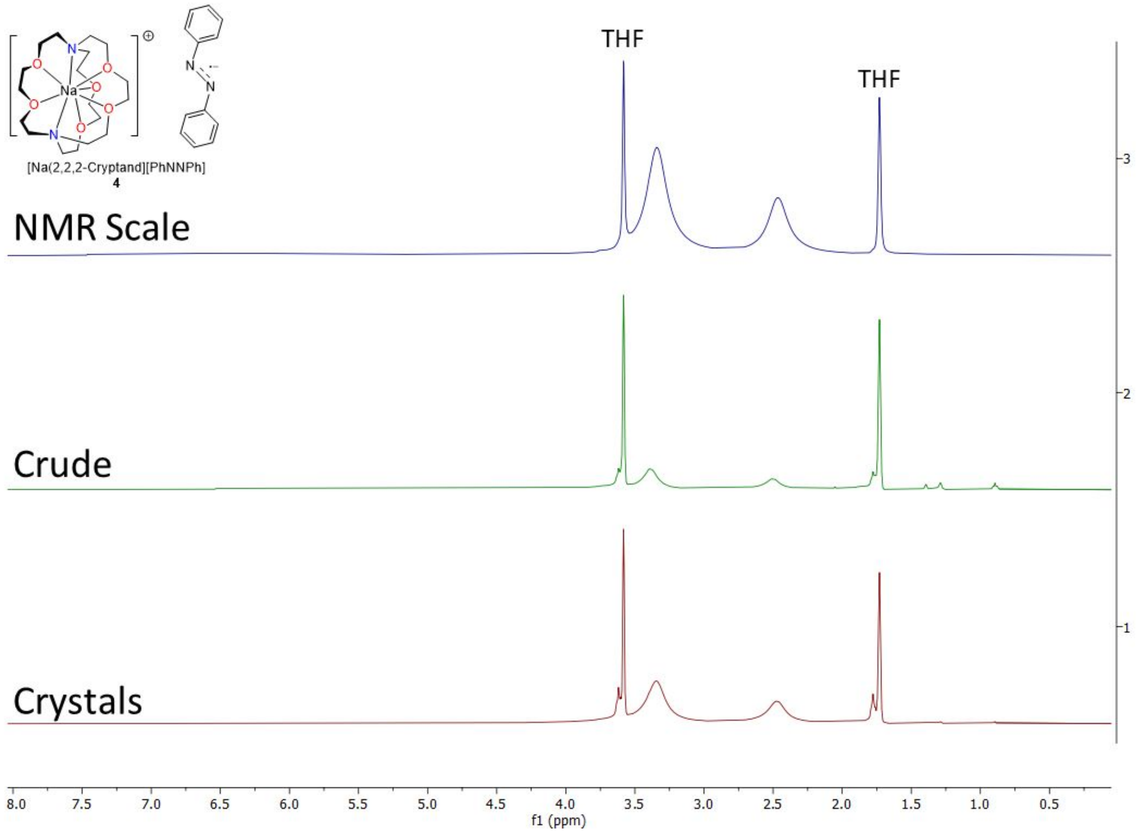

Figure S19: Stacked ^{1}H NMR (400 MHz, 25 °C) of **4** in d_8-THF. Blue: NMR scale reaction; green: crude product; red: single crystals.

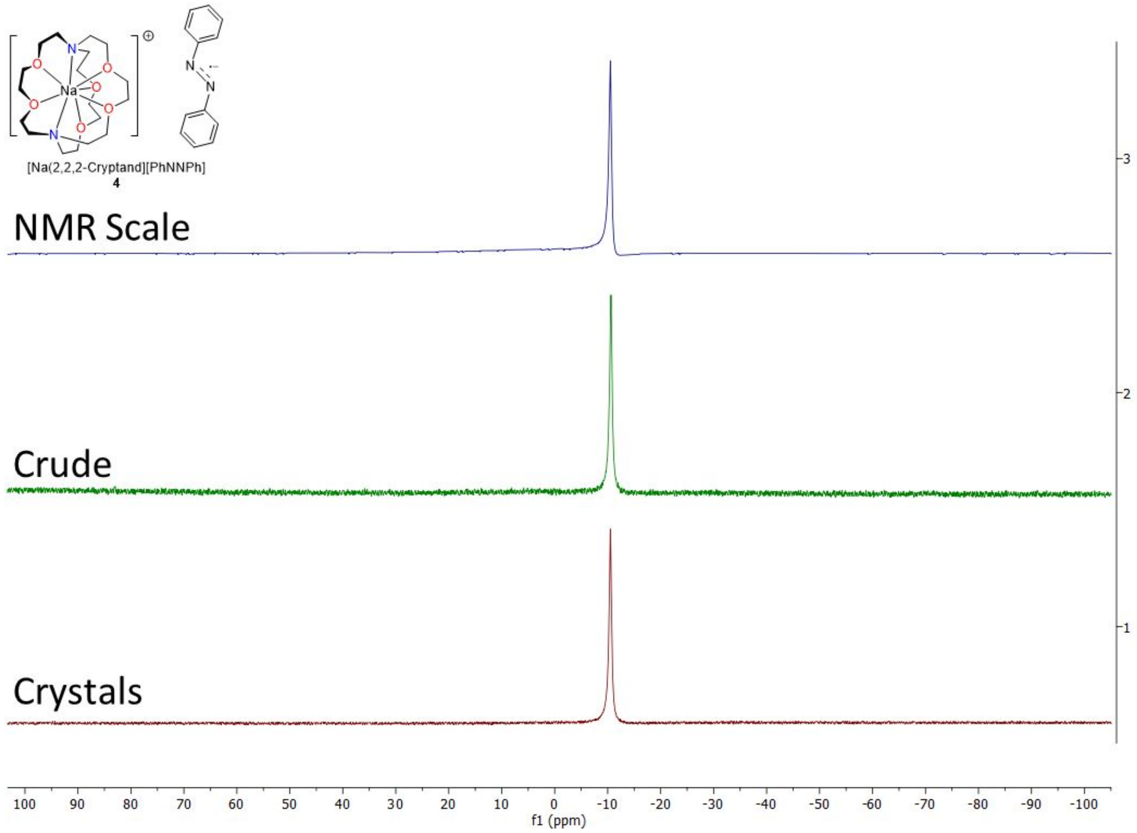

Figure S20: Stacked ^{23}Na NMR (106 MHz, 25 °C) of **4** in d_8-THF. Blue: NMR scale reaction; green: crude product; red: single crystals.

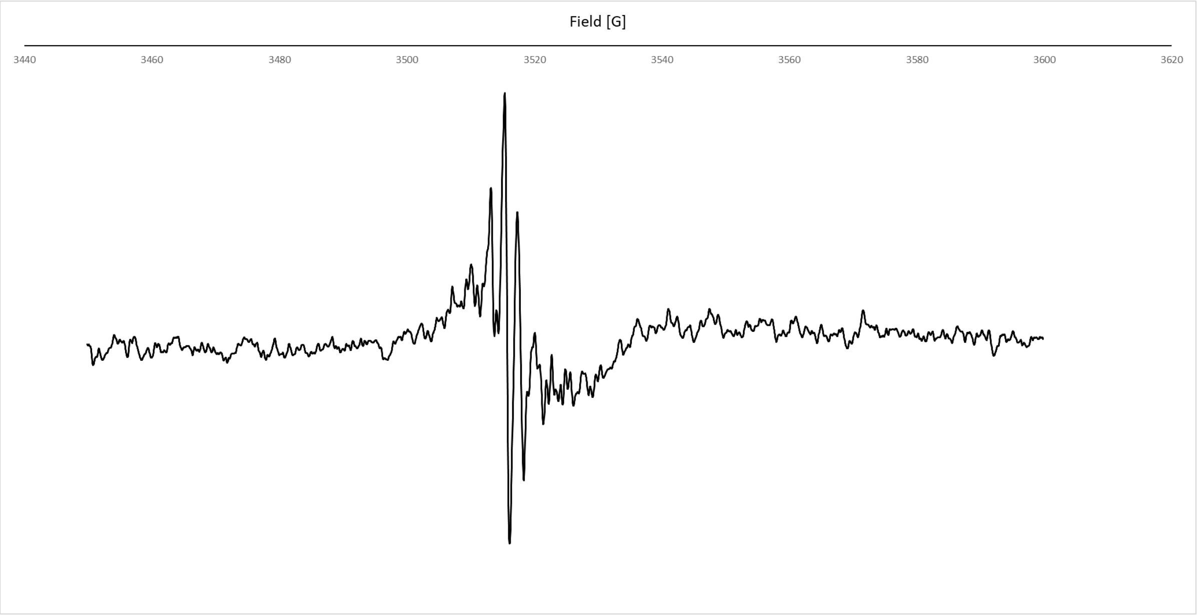

Figure S21: An EPR spectrum (X-band CW, 25 °C) of **4** in THF solution.

1.6 Single Crystal X-Ray Diffraction

General Procedure:

Single crystal diffraction data were collected on a XtaLAB Synergy HyPix-Arc 100 diffractometer using copper radiation ($\lambda_{CuK\alpha}$ = 1.54184 Å). Data were collected at 150 K using an Oxford Cryosystems CryostreamPlus open-flow N_2 cooling device. Intensities were corrected for absorption using a multifaceted crystal model created by indexing the faces of the crystal for which data were collected[1]. Cell refinement, data collection and data reduction were undertaken via the software CrysAlisPro [2].

All structures were solved using XT [3] and refined by XL [4] using the Olex2 interface [5]. All non-hydrogen atoms were refined anisotropically and hydrogen atoms were positioned with idealised geometry. The displacement parameters of the hydrogen atoms were constrained using a riding model with $U_{(H)}$ set to be an appropriate multiple of the U_{eq} value of the parent atom.

SCXRD data tables

Table S1. Crystal Data and Refinement Details for [Na^+(2,2,2-cryptand)Na^-] (**1**) in *I*2 as in this work.

Empirical formula	$C_{18}H_{36}N_2Na_2O_6$
Formula weight	422.47
Temperature/K	150.0(2)
Crystal system	monoclinic
Space group	I2
a/Å	13.9517(6)
b/Å	8.7304(3)
c/Å	10.8769(5)
α/°	90
β/°	106.010(4)
γ/°	90
Volume/Å^3	1273.46(9)
Z	2
ρ_{calc}g/cm^3	1.102
μ/mm^{-1}	0.957
F(000)	456.0
Crystal size/mm^3	0.31 × 0.15 × 0.03
Radiation	CuKα (λ = 1.54184)
2Θ range for data collection/°	9.18 to 154.07
Index ranges	$-16 \leq h \leq 17, -9 \leq k \leq 10, -13 \leq l \leq 13$
Reflections collected	11835
Independent reflections	2400 [R_{int} = 0.0270, R_{sigma} = 0.0168]
Data/restraints/parameters	2400/1/210
Goodness-of-fit on F^2	1.077
Final R indexes [I>=2σ (I)]	R_1 = 0.0248, wR_2 = 0.0741
Final R indexes [all data]	R_1 = 0.0257, wR_2 = 0.0748
Largest diff. peak/hole / e Å^{-3}	0.14/-0.16
Flack parameter	0.06(4)

Table S2. Crystal Data and Refinement Details for [Na^+(2,2,2-cryptand)Na^-] (**1**) in $R3_2$ as reported in 1974 by Dye et al. (*J. Am. Chem. Soc.* **1974**, *96*, 608-609), CSD reference CRYPNA10.

Empirical formula	$C_9H_{18}N_3NaO$
Formula weight	207.25
Temperature/K	150.0(2)
Crystal system	trigonal
Space group	$R3_2$
a/Å	8.7415(2)
b/Å	8.7415(2)
c/Å	28.9149(9)
α/°	90
β/°	90
γ/°	120
Volume/Å^3	1913.48(11)
Z	6
ρ_{calc}g/cm^3	1.079
μ/mm^{-1}	0.873
F(000)	672.0
Crystal size/mm^3	0.33 × 0.15 × 0.03
Radiation	CuKα (λ = 1.54184)
2Θ range for data collection/°	9.176 to 153.644
Index ranges	$-11 \le h \le 9$, $-9 \le k \le 10$, $-35 \le l \le 35$
Reflections collected	11844
Independent reflections	894 [R_{int} = 0.0330, R_{sigma} = 0.0106]
Data/restraints/parameters	894/48/71
Goodness-of-fit on F^2	1.209
Final R indexes [I>=2σ (I)]	R_1 = 0.0680, wR_2 = 0.2126
Final R indexes [all data]	R_1 = 0.0685, wR_2 = 0.2139
Largest diff. peak/hole / e Å^{-3}	0.79/-0.68
Flack parameter	0.10(4)

Table S3. Crystal Data and Refinement Details for $[Ph_3C]^-$ $[Na(2,2,2\text{-cryptand})]^+$ (**2**)

Empirical formula	$C_{78}H_{110}N_4Na_2O_{13}$
Formula weight	1357.67
Temperature/K	150.0(2)
Crystal system	monoclinic
Space group	$P2_1$
a/Å	9.7751(2)
b/Å	16.5217(5)
c/Å	22.3659(6)
α/°	90
β/°	91.800(2)
γ/°	90
Volume/$Å^3$	3610.34(16)
Z	2
$\rho_{calc}g/cm^3$	1.249
μ/mm^{-1}	0.776
F(000)	1464.0
Crystal size/mm^3	0.21 × 0.1 × 0.06
Radiation	CuKα (λ = 1.54184)
2Θ range for data collection/°	3.952 to 155.1
Index ranges	$-8 \le h \le 11$, $-20 \le k \le 20$, $-28 \le l \le 28$
Reflections collected	43193
Independent reflections	43193 [R_{int} = 0.0327, R_{sigma} = 0.0334]
Data/restraints/parameters	43193/1222/956
Goodness-of-fit on F^2	1.040
Final R indexes [I>=2σ (I)]	R_1 = 0.0381, wR_2 = 0.0930
Final R indexes [all data]	R_1 = 0.0428, wR_2 = 0.0965
Largest diff. peak/hole / e $Å^{-3}$	0.34/-0.20
Flack parameter	0.24(3)

Table S4. Crystal Data and Refinement Details for $[Ph_2CH]^-$ $[Na(2,2,2\text{-cryptand})]^+$ (**3**)

Empirical formula	$C_{31}H_{47}N_2NaO_6$
Formula weight	566.69
Temperature/K	150.0(2)
Crystal system	monoclinic
Space group	C2/c
a/Å	21.8563(16)
b/Å	8.7447(3)
c/Å	20.0160(15)
α/°	90
β/°	127.459(11)
γ/°	90
Volume/Å^3	3036.7(5)
Z	4
ρ_{calc}g/cm^3	1.240
μ/mm^{-1}	0.807
F(000)	1224.0
Crystal size/mm^3	0.14 × 0.11 × 0.02
Radiation	CuKα (λ = 1.54184)
2Θ range for data collection/°	9.478 to 154.242
Index ranges	$-26 \leq h \leq 26$, $-11 \leq k \leq 10$, $-21 \leq l \leq 24$
Reflections collected	27126
Independent reflections	3106 [R_{int} = 0.0348, R_{sigma} = 0.0175]
Data/restraints/parameters	3106/372/241
Goodness-of-fit on F^2	1.170
Final R indexes [I>=2σ (I)]	R_1 = 0.0428, wR_2 = 0.0976
Final R indexes [all data]	R_1 = 0.0470, wR_2 = 0.1006
Largest diff. peak/hole / e Å^{-3}	0.22/-0.23

Table S5. Crystal Data and Refinement Details for $[PhNNPh]^{-}$ $[Na(2,2,2\text{-cryptand})]^{+}$ (**4**)

Empirical formula	$C_{30}H_{46}N_4NaO_6$
Formula weight	581.70
Temperature/K	150.0(2)
Crystal system	monoclinic
Space group	C2/c
a/Å	22.492(2)
b/Å	8.7552(5)
c/Å	19.7484(18)
α/°	90
β/°	126.438(14)
γ/°	90
Volume/Å^3	3128.7(6)
Z	4
ρ_{calc}g/cm^3	1.235
μ/mm^{-1}	0.816
F(000)	1252.0
Crystal size/mm^3	0.17 × 0.08 × 0.06
Radiation	CuKα (λ = 1.54184)
2Θ range for data collection/°	9.498 to 153.688
Index ranges	$-23 \leq h \leq 28$, $-10 \leq k \leq 10$, $-24 \leq l \leq 24$
Reflections collected	14707
Independent reflections	2988 [R_{int} = 0.0207, R_{sigma} = 0.0163]
Data/restraints/parameters	2988/463/250
Goodness-of-fit on F^2	1.075
Final R indexes [I>=2σ (I)]	R_1 = 0.0309, wR_2 = 0.0819
Final R indexes [all data]	R_1 = 0.0356, wR_2 = 0.0848
Largest diff. peak/hole / e Å^{-3}	0.16/-0.18

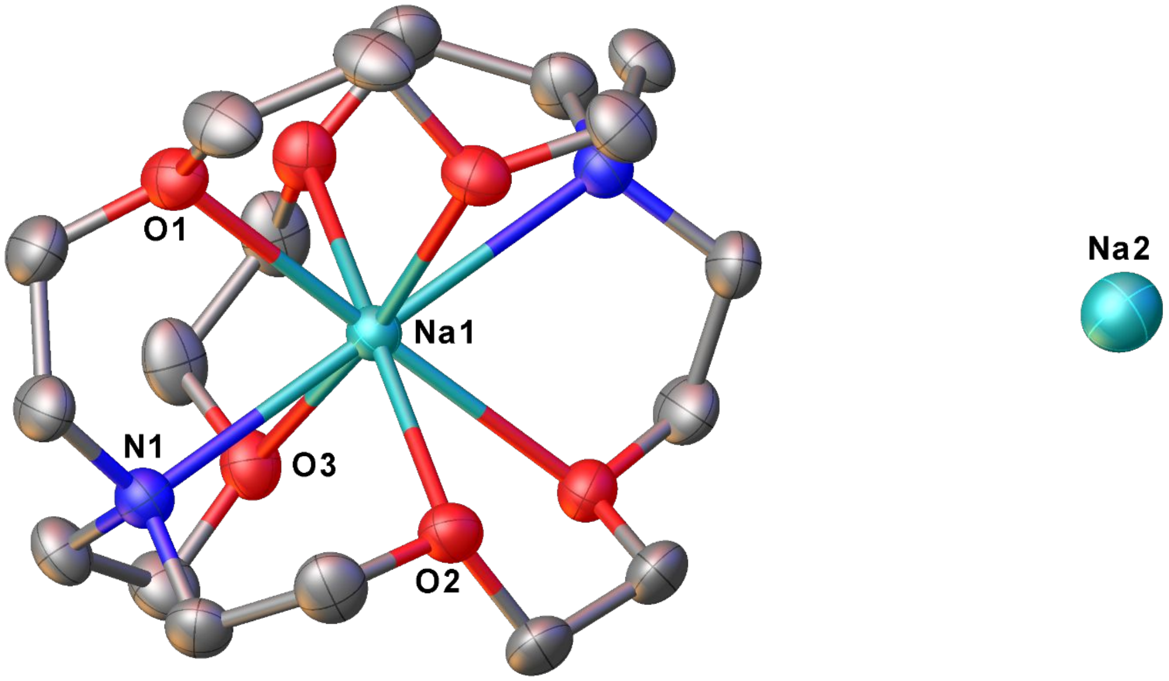

Figure S22: Crystal structure of [Na^+(2,2,2-cryptand)Na^-] (**1**). Key bond lengths (Å): Na1–O1 2.5390(15), Na1–O2 2.5393(16), Na1–O3 2.5417(10), Na1–N1 2.6650(10), Na1·····Na2 6.9732(12).

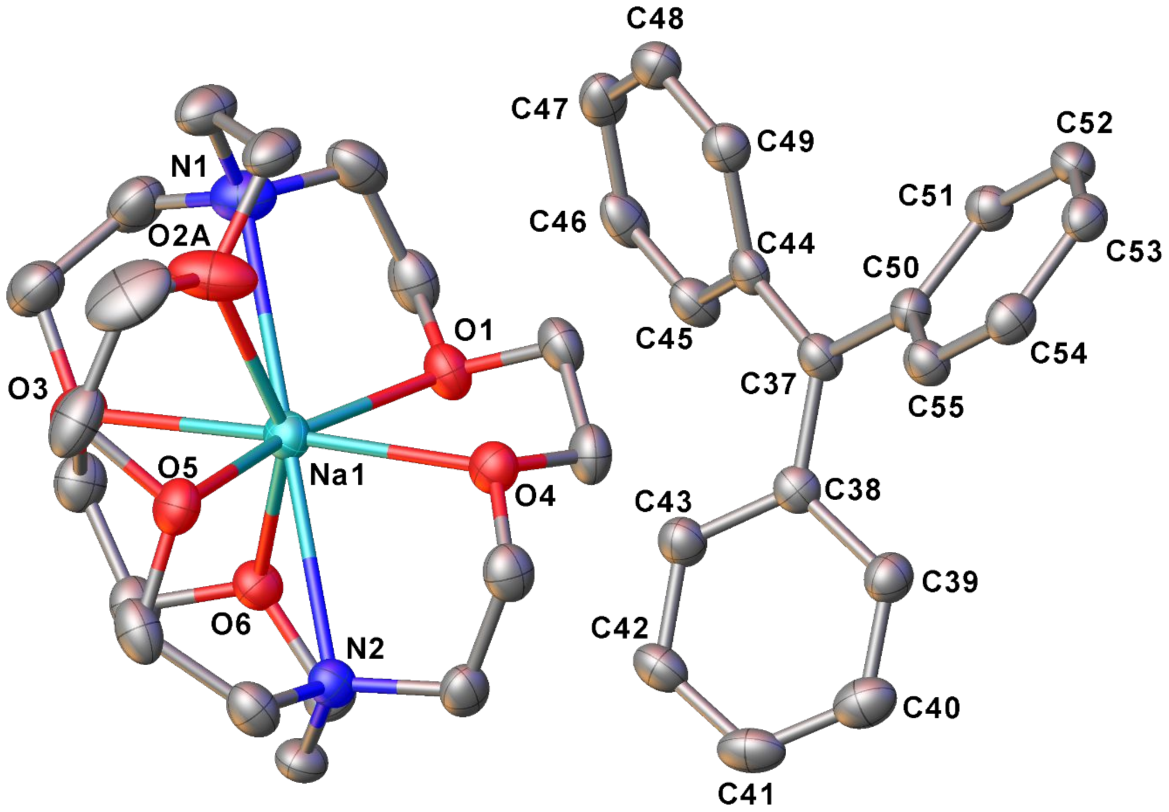

Figure S23: Crystal structure of $[Ph_3C]^{-}$ $[Na(2,2,2\text{-cryptand})]^{+}$ (**2**). Key bond lengths: Na1–O1 2.403(3), Na1–O3 2.605(3), Na1–O4 2.472(3), Na1–O5 2.598(3), Na1–O6 2.410(3), Na1–N1 2.854(4), Na1–N2 2.858(4), C37–C38 1.455(6), C37–C44 1.442(5), C37–C50 1.462(6), C38–C39 1.425(5), C38–C43 1.414(6), C39–C40 1.378(6), C40–C41 1.381(7), C41–C42 1.382(6), C42–C43 1.383(6), C44–C45 1.421(6), C44–C49 1.421(6), C45–C46 1.380(5), C46–C47 1.398(6), C47–C48 1.382(6), C48–C49 1.379(5), C50–C51 1.418(6), C50–C55 1.414(5), C51–C52 1.380(6), C52–C53 1.384(5), C53–C54 1.389(6), C54–C55 1.377(6).

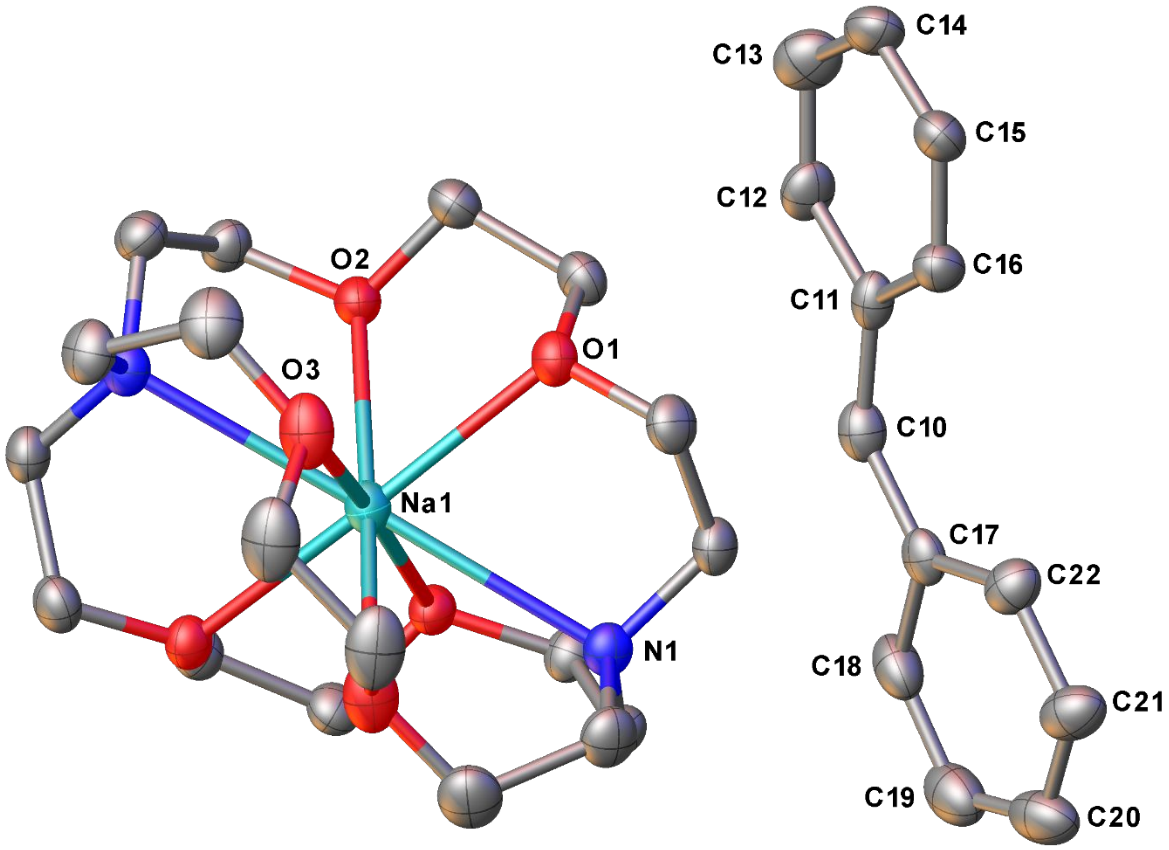

Figure S24: Crystal structure of $[Ph_2CH]^-$ $[Na(2,2,2\text{-cryptand})]^+$ (**3**). Key bond lengths (Å): Na1–O1 2.3835(11), Na1–O2 2.5670(13), Na1–O3 2.5607(14), Na1–N1 2.7505(13), C10–C11 1.423(7), C10–C17 1.414(7), C11–C12 1.417(7), C11–C16 1.408(5), C12–C13 1.378(8), C13–C14 1.393(10), C14–C15 1.393(8), C15–C16 1.389(5), C17–C18 1.412(6), C17–C22 1.409(6), C18–C19 1.386(10), C19–C20 1.392(10), C20–C21 1.400(7), C21–C22 1.392(5),

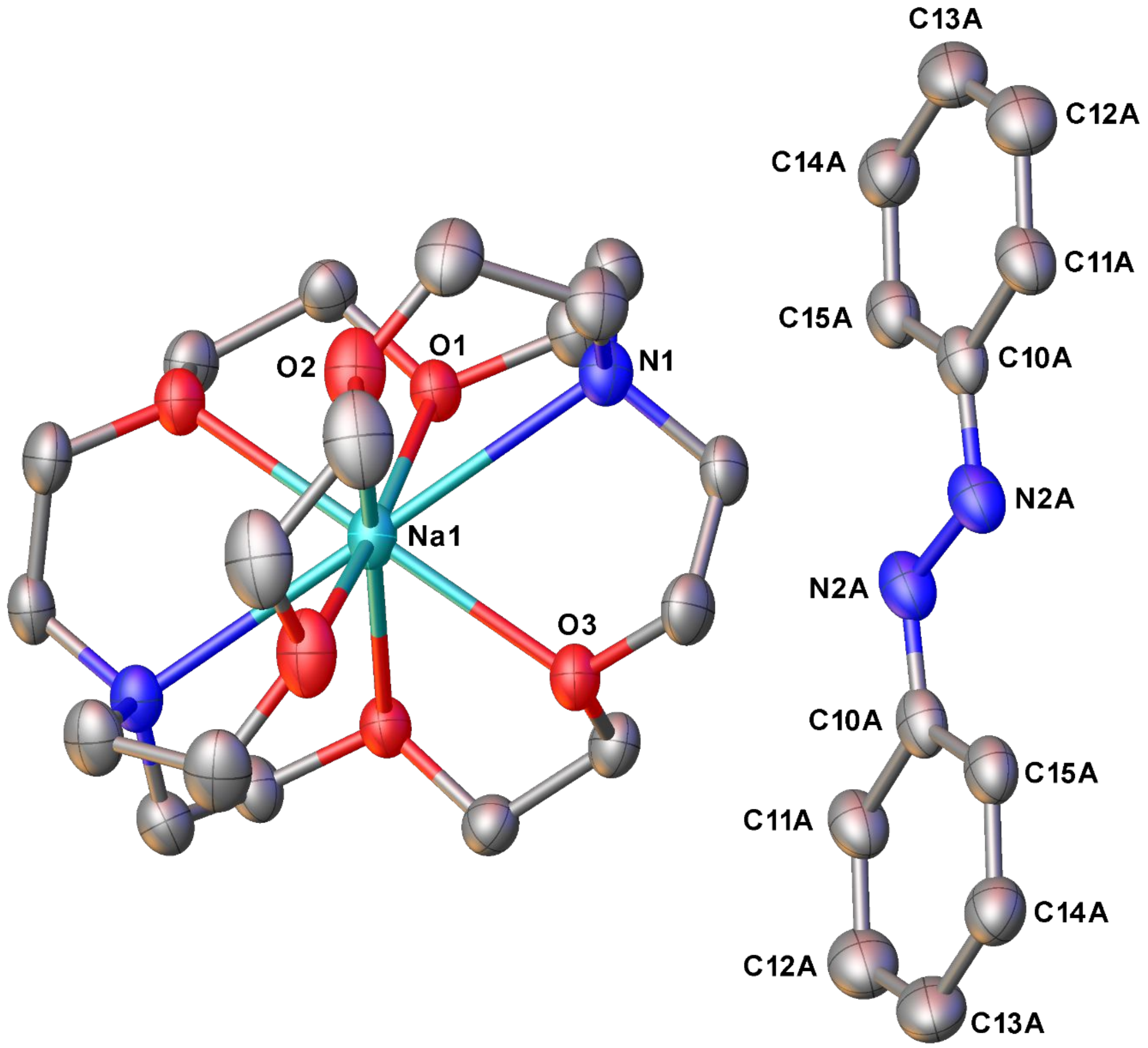

Figure S25: Crystal structure of [PhNNPh]$^{-}$ [Na(2,2,2-cryptand)]$^{+}$ (**4**). Key bond lengths (Å): Na1–O1 2.5907(9), Na1–O2 2.5645(10), Na1–O3 2.3815(8), Na1–N1 2.7539(10), N2A–N2A2 1.352(3), N2A–C10A 1.373(3), C10A–C11A 1.408(3), C10A–C15A 1.415(3), C11A–C12A 1.372(4), C12A–C13A 1.396(4), C13A–C14A 1.401(3), C14A–C15A 1.385(3).

Section 2. Computational details and data

2.1 General Procedure:

Geometry optimisation and vibrational frequency calculations were completed for each structure considered in this work; inputs were created using experimental SCXRD structures where appropriate. The calculations utilised the hybrid DFT functional B3LYP along with a 6-311+g** basis set for all atoms [7] [8], in the case of molecules that possessed spin unrestricted B3LYP was used. The effects of THF molecules as a solvent were included through the implementation of the integral equation formulation of the polarizable continuum model (IEFPCM) and Grimme's D3 dispersion correction with Becke-Johnson damping was used as implemented in Gaussian 16 [9]. Each structure optimised in this work was confirmed to be a minimum on the potential energy surface, through observation of 3N-6 real vibrational frequencies. The geometry optimisation, vibrational frequency, natural bond orbital charge analysis and NMR shielding tensor calculations utilised in this work were all completed using the Gaussian 16 Rev. C.01 package. Molecular orbital diagrams and spin density orbital diagrams were generated using ChemCraft 1.8 based on the relevant optimised geometry from the Gaussian 16 calculation [9] [10] [11].

2.2 Gibbs free energy of formation calculations:

In order to assess the thermodynamic feasibility of the 1-electron and 2-electron reaction routes for the reaction between **1** and tritylamine the Gibbs free energy of formation for each process was investigated.

The electronic energy (EE), thermal correction to Gibbs free energy (ΔG_{corr}) and thermally corrected Gibbs free energy ($\Delta G_{(298)}$) for each species were obtained from the calculations and are shown in Table S3 alongside the sum of the $\Delta G_{(298)}$ for the reactants and both sets of products.

Table S6. The EE, ΔG_{corr}, $\Delta G_{(298)}$ and the sum of $\Delta G_{(298)}$ for the reactants and both sets of products for the reaction between **1** and tritylamine.

Structure	EE / $kJmol^{-1}$	ΔG_{corr} / $kJmol^{-1}$	$\Delta G_{(298)}$ / $kJmol^{-1}$	$\Sigma(\Delta G_{(298)})$ / $kJmol^{-1}$
Reactants				
**1** ([Na(2,2,2-cryptand)]$^+$Na$^-$)	-4183683.0528	1303.3271	-4182379.7546	-6253999.2598
tritylamine	-2072305.6797	686.1692	-2071619.5053	
1-electron reaction route products				
[Na(2,2,2-cryptand)]$^+$ [Ph_3CNH]$^-$	-5828335.3727	2020.8368	-5826314.5254	-6253803.2111
H radical	-1318.4597	-27.9721	-1346.4325	
Na(0)	-426102.6345	-39.6004	-426142.2532	
2-electron reaction route products				
**2** ([Na(2,2,2-cryptand)]$^+$ [Ph_3C]$^-$)	-5683026.8127	1981.7642	-5681045.0853	-6254225.1054
$NaNH_2$	-573172.1961	-7.8240	-573180.0201	

The Gibbs free energy of formation (ΔG_f) for the two potential reaction routes was calculated using Equation S1, yielding values of 196 and -226 $kJmol^{-1}$ for the 1 and 2-electron reaction routes respectively (rounded to the nearest whole number).

$$\Delta G_f = \sum \Delta G_{(298)}(products) - \sum \Delta G_{(298)}(reactants)$$

Equation S1.

A similar approach was attempted for the reaction of **1** with azobenzene, however, the hypothesised product of the 2-electron reaction (uranium complex analogue) did not appear to resemble a minimum energy structure. Each attempt to optimise the hypothesised product resulted in breakage of the 4-membered ring structure in the centre of the complex, suggesting that the product itself is not feasible.

2.3 Molecular orbital diagrams of **1**, **2**, **4**

The following molecular orbital diagrams are based on the optimised minimum structures of **1**, **2** and **4** are show in Figures S11-13 respectively.

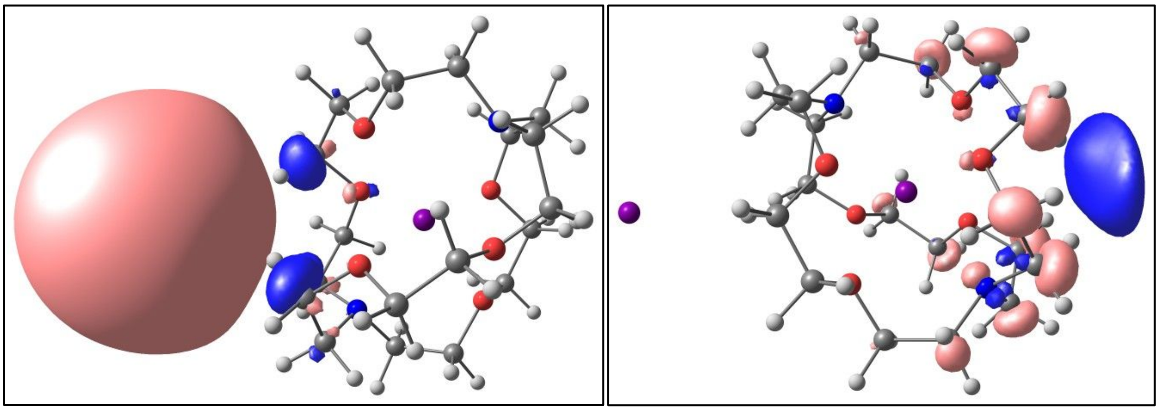

Figure S26: Diagrams of the HOMO (left) and LUMO (right) of the optimised structure of **1** generated using Chemcraft 1.8 with a contour value of 0.02**.**

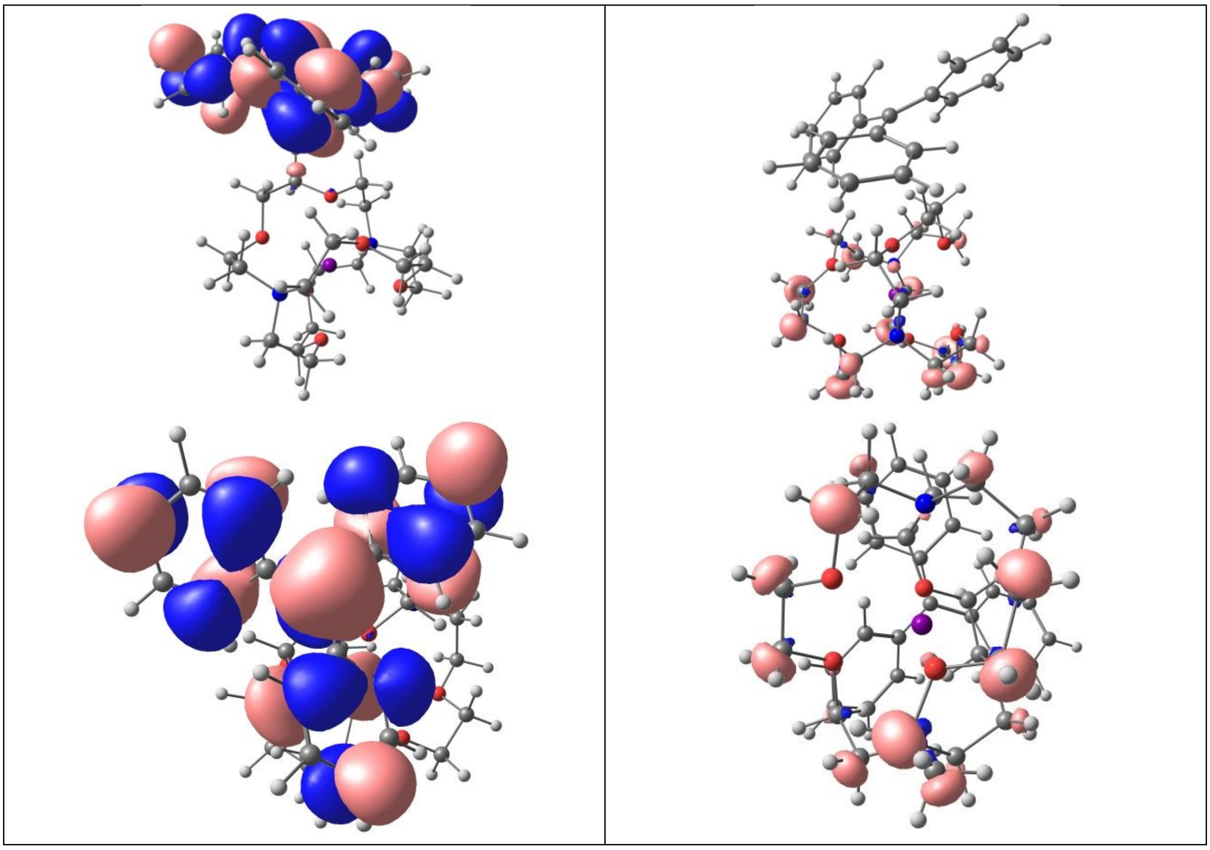

Figure S27: Diagrams of the HOMO (left) and LUMO (right) of the optimised structure of **2** generated using Chemcraft 1.8 with a contour value of 0.02**.**

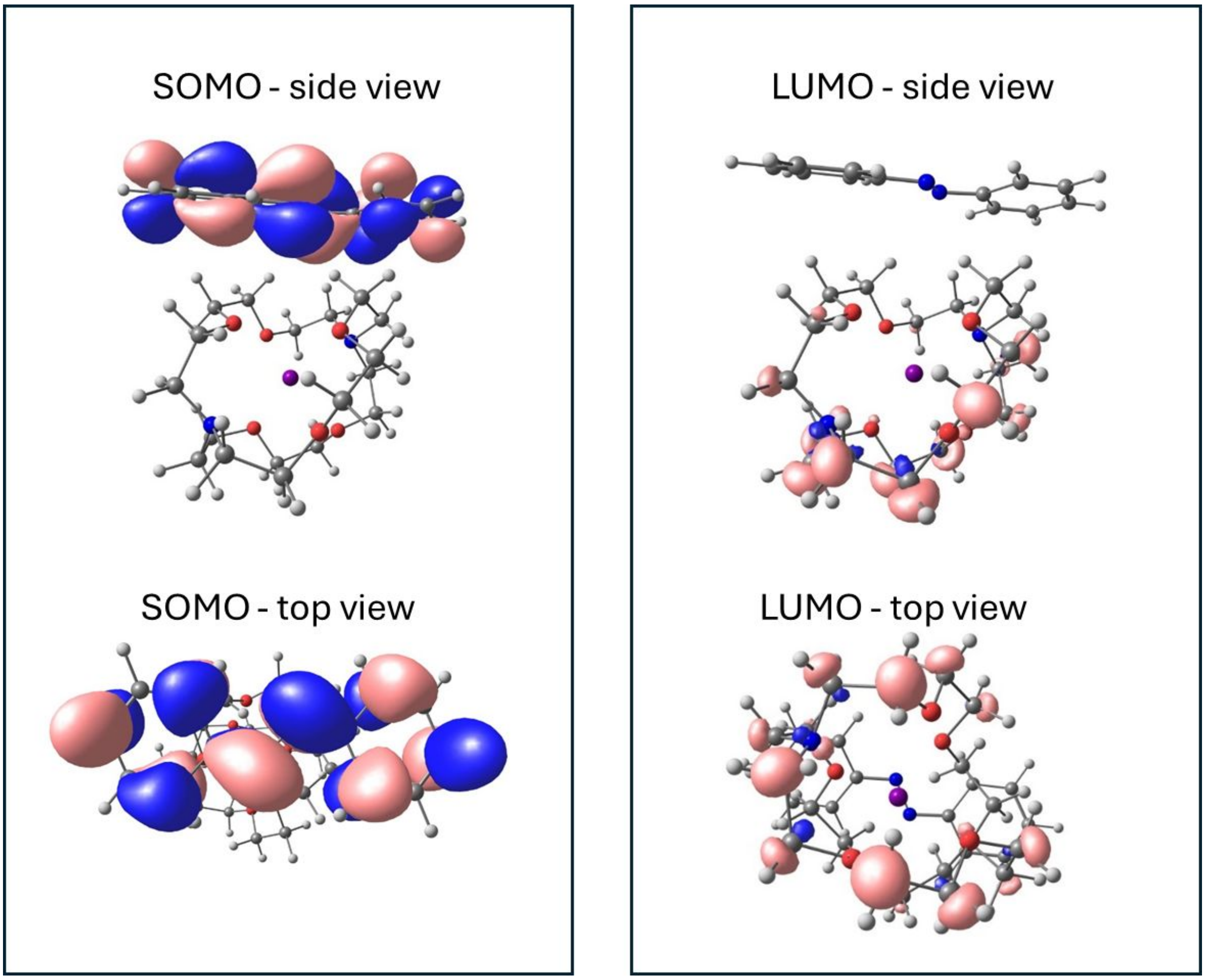

Figure S28: Diagrams of the α highest energy SOMO (left - with both side and top view) and LUMO (right -with both a side and top view) of **4** generated using Chemcraft 1.8 with a contour value of 0.02**.**

2.4 Spin density analysis of **4**

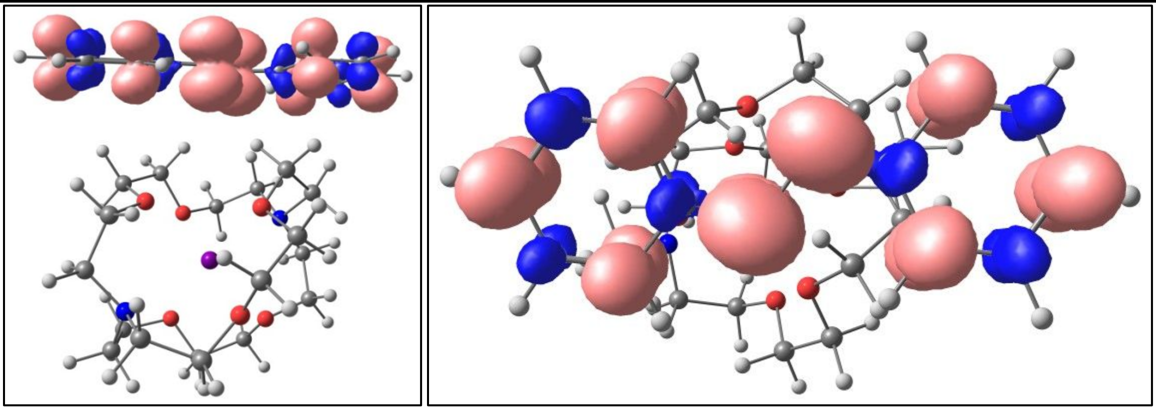

Figure S29: Diagrams of the spin density orbitals of **4** from both a side view (left) and a top view (right), images generated using Chemcraft 1.8 with a contour value of 0.002**.**

As can be seen from the spin density plots, the unpaired electron is delocalised across the entire [Ph-N-N-Ph] moiety with the largest lobes present on the two central N atoms (28 % likelihood of being located on either of the N atoms).

Section 3. References

[1] R. C. Clark, J.S. Reid, The analytical calculation of absorption in multifaceted crystals. *Acta Cryst.,* 1995, A**51**, 887

[2] CrysAlisPro, Rigaku Oxford Diffraction, Tokyo, Japan.

[3] Sheldrick, G.M. SHELXT – Integrated space-group and crystal-structure determination. *Acta Crystallogr., Sect. A: Found. Crystallogr.* **2015,** *71*, 3-8.

[4] Sheldrick, G.M. A short history of SHELX. *Acta Crystallogr., Sect. A: Found. Crystallogr.* **2008,** *64*, 112-122.

[5] Dolomanov, O.V.; Bourhis, L.J.; Gildea, R.J.; Howard, J.A.K.; Puschmann, H. OLEX2: a complete structure solution, refinement and analysis program. *J. Appl. Cryst.* **2009,** *42*, 339-341.

[6] Becke, A. D. Density-functional thermochemistry. III. The role of exact exchange. *J. Chem. Phys.* **1993**, *98*, 5648.

[7] Lee, C. T.; Yang, W. T.; Parr, R. G. Development of the Colle-Salvetti correlation-energy formula into a functional of the electron density. *Phys. Rev. B* **1988**, *37*, 785.

[8] Gaussian 16, Revision C.01, M. J. Frisch, G. W. Trucks, H. B. Schlegel, G. E. Scuseria, M. A. Robb, J. R. Cheeseman, G. Scalmani, V. Barone, G. A. Petersson, H. Nakatsuji, X. Li, M. Caricato, A. V. Marenich, J. Bloino, B. G. Janesko, R. Gomperts, B. Mennucci, H. P. Hratchian, J. V. Ortiz, A. F. Izmaylov, J. L. Sonnenberg, D. Williams-Young, F. Ding, F. Lipparini, F. Egidi, J. Goings, B. Peng, A. Petrone, T. Henderson, D. Ranasinghe, V. G. Zakrzewski, J. Gao, N. Rega, G. Zheng, W. Liang, M. Hada, M. Ehara, K. Toyota, R. Fukuda, J. Hasegawa, M. Ishida, T. Nakajima, Y. Honda, O. Kitao, H. Nakai, T. Vreven, K. Throssell, J. A. Montgomery, Jr., J. E. Peralta, F. Ogliaro, M. J. Bearpark, J. J. Heyd, E. N. Brothers, K. N. Kudin, V. N. Staroverov, T. A. Keith, R. Kobayashi, J. Normand, K. Raghavachari, A. P. Rendell, J. C. Burant, S. S. Iyengar, J. Tomasi, M. Cossi, J. M. Millam, M. Klene, C. Adamo, R. Cammi, J. W. Ochterski, R. L. Martin, K. Morokuma, O. Farkas, J. B. Foresman, and D. J. Fox, Gaussian, Inc., Wallingford CT, 2016.

[9] Glendening, E. D.; Reed, A. E.; Carpenter, J. E.; Weinhold, F. NBO Version 3.1, TCI, University of Wisconsin, Madison, 1998.

[10] Chemcraft - graphical software for visualization of quantum chemistry computations. Version 1.8, build 682. https://www.chemcraftprog.com
